# From egg to slaughter: monitoring the welfare of Nile tilapia, *Oreochromis niloticus*, throughout their entire life cycle in aquaculture

**DOI:** 10.3389/fvets.2023.1268396

**Published:** 2023-09-21

**Authors:** Ana Silvia Pedrazzani, Nathieli Cozer, Murilo Henrique Quintiliano, Camila Prestes dos Santos Tavares, Vilmar Biernaski, Antonio Ostrensky

**Affiliations:** ^1^Wai Ora—Aquaculture and Environmental Technology Ltd., Curitiba, State of Paraná, Brazil; ^2^Graduate Program in Animal Science, Federal University of Paraná, Curitiba, State of Paraná, Brazil; ^3^Integrated Group of Aquaculture and Environmental Studies (GIA), Department of Animal Science, Agricultural Sciences Sector, Federal University of Paraná, Curitiba, State of Paraná, Brazil; ^4^FAI Farms, Londrina, State of Paraná, Brazil; ^5^Graduate Program in Zoology, Federal University of Paraná, Curitiba, State of Paraná, Brazil

**Keywords:** grow-out, larviculture, fish welfare, welfare indices, protocols, sustainable aquaculture

## Abstract

The primary aim of this study was to comprehensively evaluate the welfare of Nile tilapia *(Oreochromis niloticus)* throughout their entire life cycle within aquaculture, spanning from reproduction to slaughter. The methodology was structured to identify welfare indicators closely aligned with the principles of animal freedoms defined by the Farm Animal Council, encompassing environmental, health, nutritional, behavioral, and psychological freedom. Notably, psychological freedom was inherently considered within the behavioral and physical analyses of the animals. To accomplish this, an integrative systematic literature review was conducted to define precise indicators and their corresponding reference values for each stage of tilapia cultivation. These reference values were subsequently categorized using a scoring system that assessed the deviation of each indicator from established ideal (score 1), tolerable (score 2), and critical (score 3) ranges for the welfare of the target species. Subsequently, a laboratory experiment was executed to validate the pre-selected health indicators, specifically tailored for the early life stages of tilapia. This test facilitated an assessment of the applicability of these indicators under operational conditions. Building on the insights gained from this experimentation, partial welfare indices (PWIs) were computed for each assessed freedom, culminating in the derivation of a general welfare index (GWI). Mathematical equations were employed to calculate these indices, offering a quantitative and standardized measure of welfare. This approach equips tilapia farmers and processors with the tools necessary for the continuous monitoring and enhancement of their production systems and stimulate the adoption of more sustainable and ethical practices within the tilapia farming.

## 1. Introduction

The international scientific community's recent recognition of fish as sentient beings (1–4) has encouraged various countries to implement norms and regulations and enact laws to protect these animals when commercially farmed in captivity (5–8). Simultaneously, multiple actors in the food sector—such as importers, retail chains, restaurants, and their respective representative entities—began to request sustainability and animal welfare certificates as a prerequisite for purchasing processed or unprocessed aquaculture products (9, 10). This transformation has occurred in harmony with the aquaculture sector's recognition of the relevance of launching marketing and advertising campaigns focussed on animal welfare and integrating them into the industry's main collective corporate social responsibility commitments (11, 12). As a result, albeit at an early stage, the issue of “fish welfare” is gradually being incorporated into economic actors' social attitudes and practices (13).

However, many challenges must be overcome for welfare to become an inseparable element of farmed fish production. The international recommendations and guidelines currently focus on animal transport and slaughter stages and establish only the minimum animal protection standards (7, 8, 14, 15). In this way, the strict aspects of each species and the critical points in the welfare of these animals end up being neglected. The lack of scientifically based information on tested, standardized, and validated instruments for each farmed fish species and restrictions on their applicability in field situations are often cited as some of the reasons to explain these gaps (13, 16).

Amongst the ~350 species of fish farmed in aquaculture worldwide (17), tilapia, in particular, have shown significant volume growth. According to recent FAO data (18), Nile tilapia, *Oreochromis niloticus*, is currently amongst the three most farmed fish species globally, with China, Indonesia, Egypt, Bangladesh, and Brazil emerging as the largest producers (19). Aquaculture sectors have been under pressure to produce more with fewer resources—increasing production using less feed, water, and space to meet the growing global demand for fish proteins (20). However, this pressure tends to affect the environment and the welfare of farmed fish (21). In this context, the assessment of the health and welfare of tilapia becomes an increasingly relevant challenge in a global scenario. That is why these issues emerge as a central focus in the search for the development of management alternatives aimed at improving the quality of life of the animals and the quality of the final product made available by the industries to their consumers (9, 22).

The first protocol for assessing tilapia welfare, developed by Pedrazzani et al. (23) was limited to the grow-out phase of Nile tilapia. In the present study, our goal is to review the indicators proposed in that protocol, in addition to identifying and validating specific health, environmental, nutritional, and behavioral indicators for all other phases of the development cycle of *O. niloticus* in captivity, including the stages of reproduction, nursery, and transport. This approach will, for the first time, enable a more comprehensive, accurate, and personalized analysis of the evaluation and promotion of tilapia welfare throughout its entire production cycle rather than just the final grow-out stage. Moreover, in the present study, we also applied a method to establish quantitative and standardized welfare indices for each tilapia cultivation phase.

## 2. Materials and methods

### 2.1. Organization of the welfare protocol for tilapia into categories

The operational welfare indicators for tilapia were organized according to four of the five freedoms for animals established by the Farm Animal Council (24): environmental, health, nutritional, and behavioral. The fifth freedom, psychological, is intrinsically evaluated through the behavioral and physical analysis of the animals. The methodology employed followed the same principles already used in the creation of the protocols previously developed by our group for *O. niloticus* (23) and grass carp, *Ctenopharyngodon idella* (25), during the grow-out phase of both and also for white-leg shrimp, *Penaeus vannamei* throughout its entire production cycle (26).

### 2.2. Systematic review for the definition of indicators and their respective reference values

**Figure 1:**
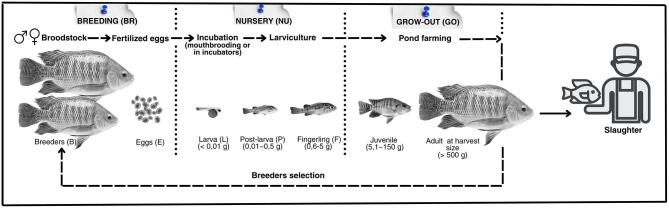
Cultivation stages (breeding, nursery, and grow-out) and development stages (breeders, eggs, larvae, post-larvae, fry, juveniles, and adults) of Nile tilapia, *Oreochromis niloticus*.

An integrative systematic review (27) was conducted using the Google Scholar platform as the research base. The aspects related to the environment, health, nutrition, and behavior associated with the species, as well as the specific welfare indicators and their respective reference values for each cultivation stage (Figure 1), were studied and defined. For the grow-out phase of tilapia, emphasis was given to cultivations carried out in earth ponds, the primary fish farming system used globally (18). The research included books, technical and scientific articles, case studies, manuals, and technical reports developed by international institutions, as well as theses and dissertations. Materials containing the following terms were selected: “*Oreochromis niloticus*” AND welfare indicator AND production stage, in the titles, abstracts, or keywords. The welfare indicators defined for each stage of the cultivation process are listed in Table 1. The search period extended from 1985 to 2023.

**Table 1:** Welfare indicators organized according to animal freedoms and production stages of Nile tilapia (BR, breeding; NU, nursery; GO, grow-out).

**Freedom**	**Indicator**	**Breeding (BR)**	**Nursery (NU)**	**Grow-out (GO)**
Environmental	Alkalinity	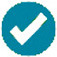	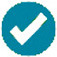	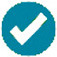
	Aquatic predators and other interspecific inhabitants	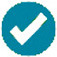	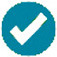	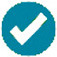
	Dissolved oxygen	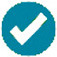	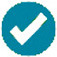	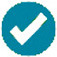
	Hapas net cleaning	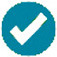		
	Nitrite	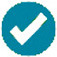	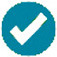	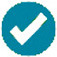
	Non-ionized ammonia	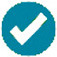	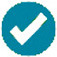	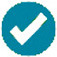
	pH	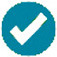	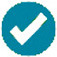	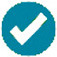
	Photoperiod	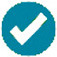	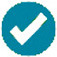	
	Sex ratio (male: female)	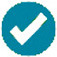		
	Temperature	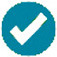	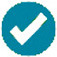	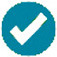
	Terrestrial predators	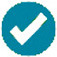		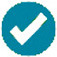
	Transparency	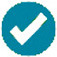		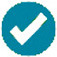
Health	Breeding control	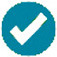		
	Conditioning/ breeding interval (days)	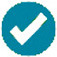		
	Eggs–macroscopical aspects	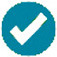		
	Emaciation state	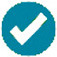	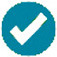	
	Eyes	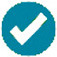	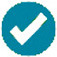	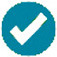
	Fins		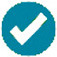	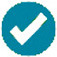
	Gills	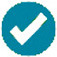		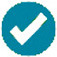
	Hatching rate		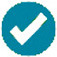	
	Invasive procedures	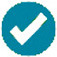		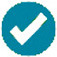
	Jaws/lips/head	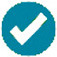	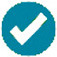	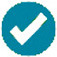
	Mortality (%)	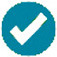	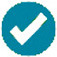	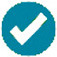
	Operculum	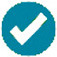	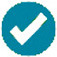	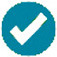
	Sexual maturation	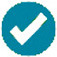		
	Skin	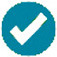	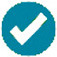	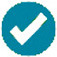
	Spine	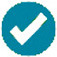	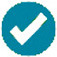	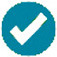
	Tail		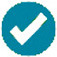	
	Yolk sac		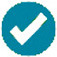	
Nutritional	Amount of feed	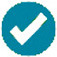	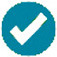	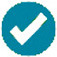
	Feed conversion ratio (FCR)			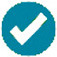
	Feed crude protein	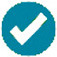	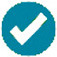	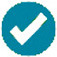
	Feeding frequency	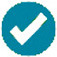	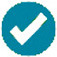	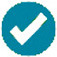
	Food distribution	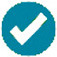	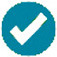	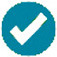
Behavioral	Anesthesia	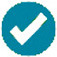		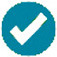
	Feed intake	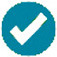	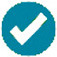	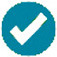
	Swimming behavior	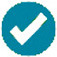	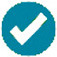	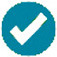
	Stunning during slaughter–reflexes			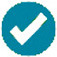

The reference values for each indicator were classified through a system involving three possible scores (1, 2, or 3). A score of 1 indicates the limits of variation of a particular indicator within the ranges considered ideal for the target species. A score of 2 pertains to variations the animals tolerate, which can cause deleterious effects, provided they are non-lethal. A score of 3 indicates significant levels of variation in a specific indicator that significantly compromises the health and even the survival of the animals, which is deemed unacceptable from an animal welfare perspective. The maximum tolerated mortality rates, the primary indicator of the degree of welfare of the fish, were set at levels much lower than those found in nature, taking into account each stage of the production cycle. The reference values for each indicator and score were established based on the literature available for the species.

### 2.3. Preliminary assessment of the indicators for the early life stages of tilapia

An experimental trial was conducted to establish health protocols for the early life stages of tilapia and to test the pre-selected indicators, allowing for the assessment of their applicability under operational conditions and the evaluation techniques for each indicator. The experiment was conducted at the Laboratory of Research with Aquatic Organisms (LAPOA) of the Integrated Group of Aquaculture and Environmental Studies (GIA) at the Federal University of Paraná, Curitiba, Brazil. All husbandry and experimental procedures were approved by the Animal Use Ethics Committee of the Agricultural Sciences Campus (CEUA) of the Federal University of Paraná (protocol number 021/2023).

**Figure 2:**
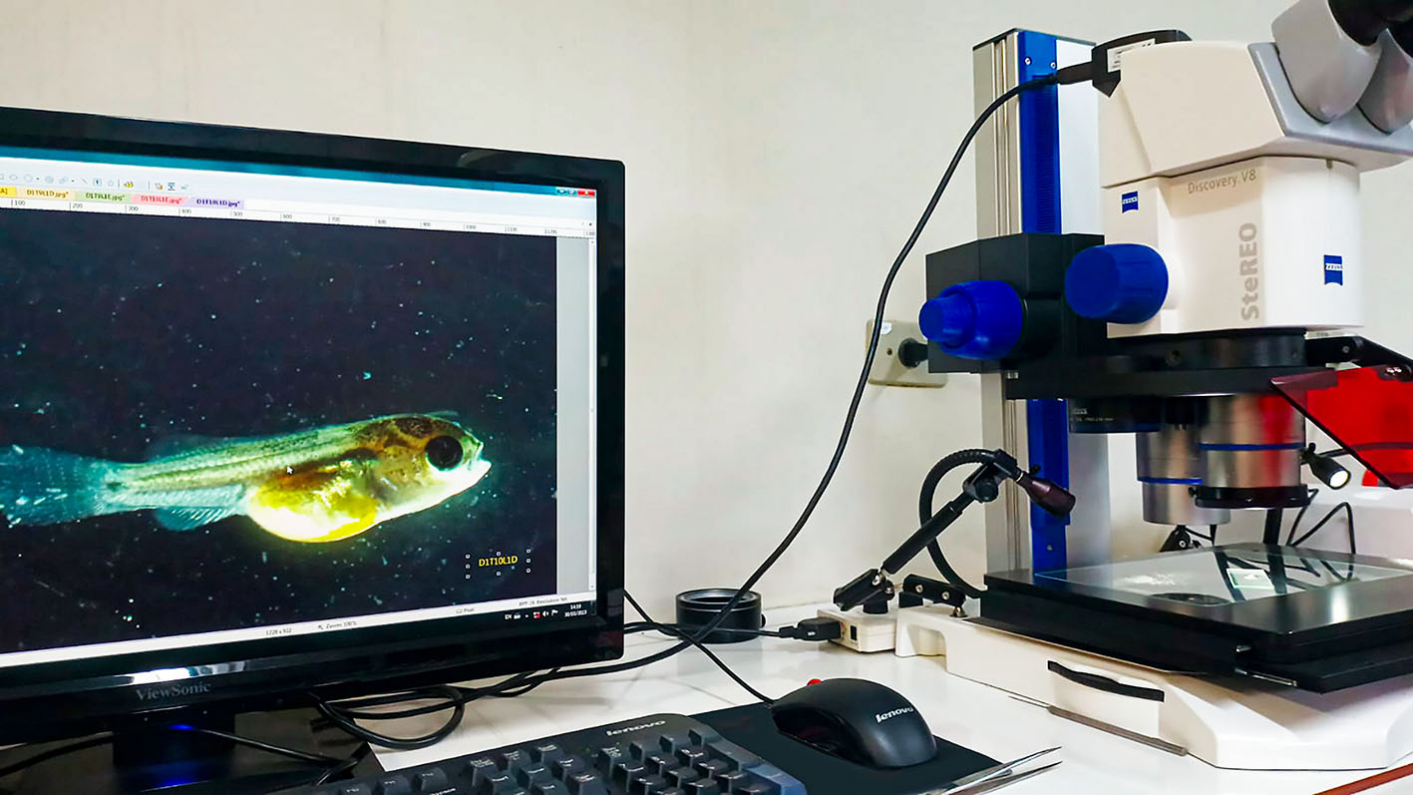
Image analysis system used to evaluate and record any health alterations in larvae and post-larvae of *Oreochromis niloticus*.

A total of 480 newly hatched tilapia larvae were used, subdivided into 12 aquariums of 30 liters in volume, each linked to a chemical–biological filtration system, under controlled conditions of temperature (27.01 ± 1.0°C), pH (7.83 ± 0.2), and dissolved oxygen (5.40 ± 0.4 mg/L). Over 15 days, a tilapia larva from each aquarium was randomly selected using a catch net and carefully and individually transferred to cell culture dishes, duly labeled, containing 10 mL of water on a daily basis. This procedure ensured that the samplings were representative, giving greater precision to the results. The collected fish were anesthetized with clove oil at a concentration of 500 mg/L and kept until they reached stage V anesthesia (~5 min). At that point, the fish have a medullary collapse and permanent unconsciousness (28). The cell culture dishes containing the collected animals were then transferred to the imaging laboratory, where the animals underwent physical evaluation procedures and photographic recording to determine their health status and evaluate the welfare indicators. The following organs were assessed under a stereo microscope (Figure 2): eyes, mouth and jaws, skin, fins, gill covers, spinal column, and yolk sac. Next, an individual and bilateral photographic record of each individual was made. The indicators that proved unfeasible to measure under operational conditions and on a commercial scale were excluded from the final version of the protocol. After this final verification, the protocols were reorganized in the format and content presented in Tables 1–10.

### 2.4. Application of welfare indices

Calculation of tilapia welfare indices utilized the same mathematical equations proposed by Pedrazzani et al. (25) for evaluating the welfare degree of *C. idella*. The weights assigned to each indicator in the respective indices were established based on the number of valid bibliographic references found for *O. niloticus* in each production phase, using Google Scholar as the research platform. The variable “Y” was calculated as the integer part of the natural logarithm (ln) of the number of articles identified through specific keywords, as shown in Equation 1. Consequently, partial welfare indices (PWIs) were proposed for each category, along with the general welfare index (GWI), calculated from the PWIs. The PWIs were computed using Equation 2, which considers the weights assigned to each indicator and their respective scores.


(1) $$\begin{array}{l} Y=INT\;\left(\text{ln}\left(n\right)\right) \end{array}$$

(2) $$\begin{array}{l} PWI_{x}=\left(\frac{\sum Y}{\sum \left(S\times Y\right)}\;\times \left(1.4925-0.4925\right)\right) \end{array}$$

where:

*PWI*: Partial welfare index standardized to vary continuously between 0 (critical risk of harm to farmed fish welfare) and 1 (maximum welfare or, otherwise, minimum risk of injury to animal welfare), regardless of the number of indicators used in each freedom.

*X*: Freedom (En, environmental; Be, behavioral; Nu, nutritional, or He, health).

*Y*: Weight assigned to the specific indicator.

*S*: Score assigned to the indicators in the analyzed fish farm.

The general welfare index (GWI) was calculated as the arithmetic mean of the PWIs, multiplied by an elimination factor (kl, Equation 3). The kl is defined based on the observed mortality rate. Thus, mortality becomes mathematically the most critical indicator for measuring the welfare degree of farmed fish in captivity. By definition, if the mortality rate exceeds 30%, the value of the elimination factor will be equal to zero (*kl* = 0), automatically indicating a “critical” classification for the GWI of the evaluated fish. If the mortality rate is below 30%, the eliminatio n factor will be equal to 1 (*kl*=1), and the welfare of the fish will be determined based on the respective indicators analyzed, their scores, and weights.


(3) $$WGI=\frac{\left((PWI_{En}+PWI_{Be}+PWI_{Nu}+PWI_{He})\times kl\right)}{4}$$

where:

*WGI*: General welfare index, que varia de 0 (critical risk of harm to farmed fish welfare) and 1 (maximum welfare or, otherwise, minimum risk of injury to animal welfare).

*kl*: Knockout level (risk of total impairment of the degree of welfare).

The partial confidence levels (CLx) proposed for each PWI are determined based on the number of indicators effectively analyzed in the field. The more indicators evaluated compared to the proposed indicators, the higher the confidence level of the results. The general confidence level (GCL) is calculated as the arithmetic mean of the CLx (Equation 5). Finally, the PWIx, GWI, CLX, and GCL are classified and interpreted according to the obtained values.


(4) $$\begin{array}{l} CL_{x}=\left(\frac{{\sum W}_{An}}{{\sum W}_{\max}}\right) \end{array}$$

*CL*_X_: PWIx confidence level.

∑*W*_*An*_: Sum of the weights of the indicators analyzed for the freedom x.

∑*W*_*max*_: Sum of the weights of all the defined indicators for the freedom x.


(5) $$GCL=\frac{\left(IR_{En}+IR_{Be}+IR_{Nu}+IR_{He}\right)}{4}$$

## 3. Results

The general protocol is divided into four categories/freedoms analyzed (environmental, health, nutritional, and behavioral), each with indicators applicable to their respective cultivation phases (Table 1).

### 3.1. Environmental welfare indicators for Nile tilapia

In the scope of environmental freedom, a set of 12 indicators has been established for different cultivation phases (Table 2). The physicochemical indicators of water have been standardized across all stages. This allows for the prevention of shocks during the transfer of animals between phases whilst maintaining strict adherence to the adopted environmental indicator reference values. Predators and aquatic and terrestrial cohabitants have also been included as environmental welfare indicators, with scores assigned based on their control or presence/absence. A score of 1 should be considered when there is no evidence of other terrestrial or aquatic species in the fishpond. In the case of terrestrial predators, a score of 2 should be applied when the fish farmer adopts control measures, such as filters or screens, but indirect contact still occurs (e.g., a visual connection between tilapia and their predators). For aquatic interspecific predators or cohabitants, a score of 2 should be applied to polyculture systems. The evaluator should assign a score of 3 when there is no control over predators or the presence of cohabitants from other species. The photoperiod should be considered a relevant indicator during the reproduction and larviculture phases. In the reproduction phase, additional indicators such as hapa cleanliness and the proportion of males and females used in tanks and fishponds were considered (Table 2).

**Table 2:** Environmental welfare reference values for different tilapia production phases (BR, breeding; NU, nursery; GO, grow-out).

**Production stages**			**Indicators**	**Scores**	**Reference values**	**References**
**BR**	**NU**	**GO**				
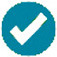	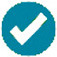	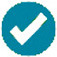	Temperature (°C)	1	24.0–31.0	(29–35)
				2	21.0–23.9 or 31.1–34.9	
				3	≤ 20.9 or ≥ 35.0	
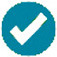	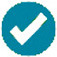	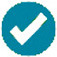	pH	1	6.0–8.5	(29, 30, 34, 36)
				2	5.5–5.9 or 8.6–9.5	
				3	≤ 5.4 or ≥ 9.6	
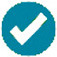	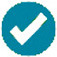	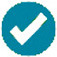	Oxygen saturation (%)	1	≥ 6	(29, 37, 38)
				2	40–59	
				3	≤ 39	
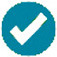	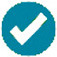	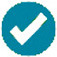	Non-ionized ammonia (mg/L of NH_3_)	1	0.00–0.05	(30, 36, 39–42)
				2	0.06–0.09	
				3	≥ 0.10	
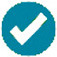	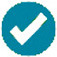	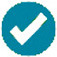	Nitrite (mg/L of NO${}_{2}^{-}$)	1	0.00–0.30	(43–47)
				2	0.31–0.49	
				3	≥ 0.50	
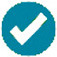	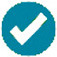	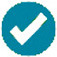	Alkalinity (mg/L of CaCO_3_)	1	30–100	(48–51)
				2	≥ 101	
				3	≤ 29	
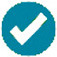	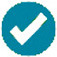	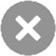	Photoperiod (Light: Dark)	1	Natural or 12L: 12D 16L:8D	(32, 39, 52–55)
				2	17L:7D−18L:6D	
				3	19L:5D or lighter; 11L:13D or darker	
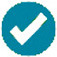	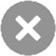	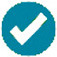	Transparency (cm)	1	30–4	(29, 48, 56)
				2	21–29 or 46–60	
				3	≤ 20 or ≥ 61	
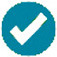	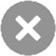	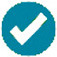	Terrestrial predators	1	Absence	(30)
				2	Controlled presence	
				3	Uncontrolled presence	
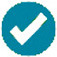	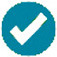	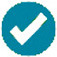	Aquatic predators and other interspecific inhabitants	1	Absence	(30)
				2	Controlled presence	
				3	Uncontrolled presence	
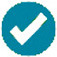	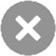	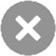	Hapas net cleaning	1	7–15	(34, 57–59)
				2	≤ 6	
				3	≥ 16	
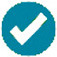	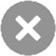	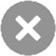	Sex ratio (male: female)	1	1:2–1:3	(30, 34, 39)
				2	1:1 or 1:4	
				3	Any other configuration	

Indicators included (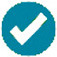) or not included (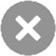) in each production phase.

### 3.2. Health welfare indicators for Nile tilapia

#### 3.2.1. Breeding (BR) and grow-out (GO) phases

Health indicators were established considering the morphological abnormalities in tilapia during their ontogenetic development, observing aspects corresponding to different life and production phases (Tables 3, 4). For breeders (BRs) and animals in the grow-out phase (GO), welfare should be assessed based on physical features such as eye appearance, jaw and lip condition, gill covers, fins, skin, gills, spine, as well as mortality rates and the conduct of invasive procedures with the fish (e.g., vaccination, microchipping, mouth cutting, and removal of dorsal spines). These procedures should be scored as 2 or 3, depending on whether anesthesia is used during execution. For breeders, other important factors for assessing welfare include the stage of sexual maturation, the interval between conditioning periods for mating, and the use of techniques to prevent inbreeding during stock formation and reproductive management. For eggs, only the macroscopic aspect was identified as a practical health indicator, with eggs that are translucent and uniform in appearance considered healthy.

**Table 3:** Health welfare reference values for tilapia breeding (BR, breeding; NU, nursery; GO, grow-out) phases.

**Production phase**			**Indicators**	**Scores**	**Description or reference values**	**References**
**BR**		**GO**				
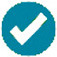	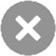	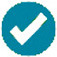	Eyes	1	Normal and healthy appearance	(39, 60, 61)
				2	Unilateral hemorrhage, exophthalmos, or traumatic injury	
				3	Bilateral bleeding, exophthalmos, or traumatic injury; chronic condition, impaired vision	
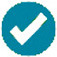	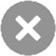	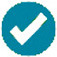	Jaw/lips	1	Normal and healthy appearance	(60, 62, 63)
				2	Mild injury or deformity (without affecting eating)	
				3	Bleeding, redness, severe injury or deformity (affecting eating)	
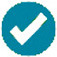	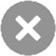	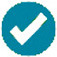	Operculum	1	Normal and healthy appearance	(63–65)
				2	Absence of tissue (< 25%)	
				3	Bleeding, redness, absence of tissue (≥ 25%)	
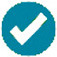	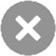	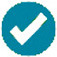	Skin	1	Normal, healthy appearance, scar tissue	(39, 66, 67)
				2	Punctual loss of scales, ulcers, or superficial lesions < 1 cm^2^	
				3	Generalized bristling or loss of scales, ulcers, or superficial lesions >1 cm^2^, redness, necrosis, darkening or lightening, bleeding, swelling, presence of parasites	
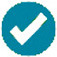	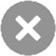	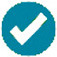	Fins	1	Normal and healthy appearance	(67–69)
				2	Scar tissue, mild necrosis, or splitting	
				3	Severe necrosis, splitting or bleeding, redness, exposure to rays, adhered foreign body, ectoparasite	
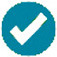	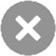	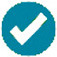	Gills	1	Normal and healthy appearance	(40, 60, 61, 66, 68, 70, 71)
				2	Light injury, mild necrosis, splitting or thickening	
				3	Bleeding, redness, pallor, severe necrosis, splitting or thickening, excess of mucus, spots, swelling, deformation, adhered foreign body, ectoparasite	
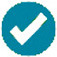	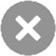	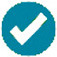	Spine	1	Normal and healthy appearance	(61, 63, 65)
				2	Light deformity (kyphosis, lordosis or scoliosis, normal body weight)	
				3	Severe deformity (kyphosis, lordosis or scoliosis, emaciation)	
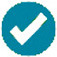	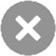	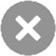	Conditioning/breeding interval (days)	1	≥10	(34, 39, 72, 73)
				2	5–9	
				3	≤ 4	
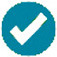	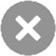	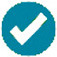	Invasive procedures	1	No invasive procedure	(23, 34, 74)
				2	Microchipping with anesthesia, mouth egg collection	
				3	Microchipping without anesthesia; mouth clipping; up-rooting of dorsal spines	
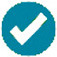	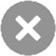	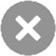	Sexual maturation	1	Mature animals. Male: Reddish colouration under the jaw; release milt when slight pressure is applied to the abdomen. Female: Ready to spawn grayish colouration under the jaw, pink to red and protruding genital papilla, opened genital pore, distended abdomen	(29)
				2	Male: do not release milt when the abdomen is pressed. Female: Pink to yellow, slightly opened genital pore, slightly distended abdomen	
				3	Male: do not release milt when the abdomen is pressed. Female: Spawned: Red genital papilla, compressed abdomen aspect or; Immature: White to clear and flat genital papilla, regular abdomen aspect	
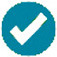	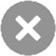	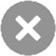	Breeding control	1	Microchipping and family physical restrain	(74, 75)
				2	Family physicals restrain without individual identification	
				3	No breeding control	
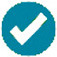	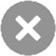	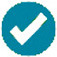	Mortality (%)	1	≤ 10	(23, 25)
				2	11–24	
				3	≥ 25	
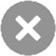	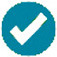	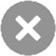	Macroscopical aspect	1	≥ 90 spherical and translucent, with yellowish colouration; the remaining with an opaque aspect	(76–79)
				2	70–89 spherical and translucent, with yellowish colouration; the remaining with an opaque aspect	
				3	≤ 69% spherical and translucent eggs and or detection of some reddish or clustered eggs; presenting white or yellow spots	

Indicators included (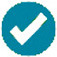) or not included (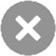) in each production phase.

**Table 4:** Health welfare reference values for tilapia nursery (NU) phase, more specifically during larvae (L), post-larvae (P), and fingerlings (F) stages.

**Stages**			**Indicators**	**Score**	**Reference values**	**References**
**L**	**P**	**F**				
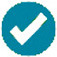	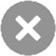	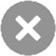	Hatching rate (% of eggs)	1	≥ 9	(77)
				2	75–89	
				3	≤ 74	
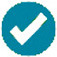	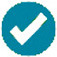	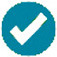	Eyes	1	Normal and healthy appearance	(40, 80–82)
				2	Unilateral: malformation or absence; exophthalmos, redness, darkening, corneal opacity, impaired vision	
				3	Bilateral: malformation or absence; exophthalmos, redness, darkening, corneal opacity, impaired vision	
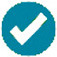	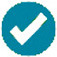	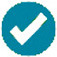	Jaws/lips/head	1	Normal and healthy appearance	(63, 83, 84)
				2	Malformation without possible feeding restriction	
				3	Malformation with possible feeding restriction, injury, ulcers, necrosis	
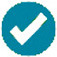	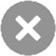	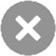	Skin	1	Fully pigmented (melanophores throughout the dorsal, ventral, and mediolateral region of the body)	(60, 63, 82, 83, 85)
				2	Partially pigmented (melanophores for some regions of the body)	
				3	Completely translucent or grayish-pale body; redness, paleness, darkening, ectoparasites, white or black spots, bleeding, swelling, ectoparasites, or increase in mucus secretion	
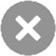	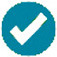	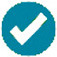	Skin	1	Normal and healthy appearance	(39, 66, 67)
				2	Scar tissue, ulcers, or superficial lesions	
				3	Severe ulcers or lesions, redness, necrosis, white or black spots, cysts, darkening or lightening, bleeding, swelling, ectoparasites, or increase in mucus secretion	
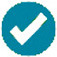	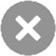	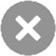	Tail	1	Normal and healthy appearance	(82, 83)
				2	Malformation without movement restriction	
				3	Malformation with movement restriction, darkening, redness	
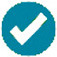	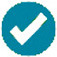	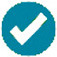	Spine	1	Functional and healthy appearance	(83)
				2	Malformation without movement restriction	
				3	Malformation with movement restriction	
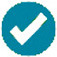	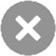	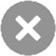	Yolk sac	1	Functional and healthy appearance	(81, 86)
				2	Malformation without size reduction	
				3	Malformation with size reduction (atrophy), hemorrhage, red spots, sub-epithelial oedema	
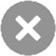	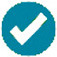	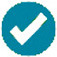	Operculum	1	Normal and healthy appearance	
				2	Malformation or lesion causing the absence of tissue (< 25% of gills covering)	(63, 84)
				3	Bleeding, swelling, redness, absence of tissue (≥ 25% of gills covering)	
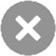	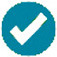	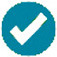	Fins	1	Functional and healthy appearance	(71, 82–85)
				2	Malformation (partial absence), ray deviations	
				3	Necrosis, redness, darkness, white spots, total absence	
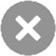	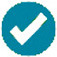	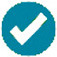	Emaciation state	1	No signs of emaciation	(83, 87)
				2	Discrete emaciation	
				3	Advanced emaciation	
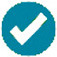	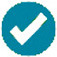	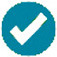	Mortality of the batch (%)	1	≤ 10	(81, 88, 89)
				2	11–15	
				3	≥ 16	

Indicators included (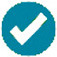) or not included (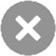) in each production phase.

#### 3.2.2. Nursery phase

The health indicators during the nursery phase were subdivided according to the ontogenetic developmental stage of the fish: larvae (L), post-larvae (PL), and fingerlings (F). The laboratory experiment proved essential for evaluating the feasibility of applying the pre-selected indicators in each phase (Table 4). The health status of the eyes, jaw/lips, and head as a whole, as well as the skin, spinal column, and fish mortality, could be easily observed with the aid of a stereoscopic loupe (10x magnification) until the animals reached ~2 cm in standard length. After that, it is possible to evaluate the external organs of the fish using only a handheld loupe with a 3x magnification capability.

The indicators exclusively adopted for the larvae were hatching rate, caudal fin formation, and yolk sac. On the other hand, gill covers and fins could only be observed in the post-larvae and fingerlings, as these structures were not fully developed in the larvae. Similarly, fish emaciation was observed only after yolk consumption i.e., in the post-larval stage. Therefore, the “emaciation” indicator was included in the protocols for assessing the welfare level of post-larvae and fingerlings. Due to the difficulty of handling fish during the early life stages and the fragility of organs during physical examination, it was impractical to evaluate the gill condition during the nursery phase. Simply manipulating the larvae during physical examination in the early days of life can cause damage to the skin, eyes, and internal organs, thereby biasing the assessment results. Thus, the convenience and feasibility of applying the protocol for tilapia larvae should be evaluated on a case-by-case basis.

### 3.3. Nutritional welfare indicators for Nile tilapia

Four relevant nutritional indicators applicable to all cultivation phases have been defined: crude protein content in the feed provided to the fish, feed quantity with the biomass of the batch, feeding frequency, and feeding distribution range in the respective cultivation system. The reference values for these indicators were determined based on the weight of tilapia (Table 5). Considering the physiological and energy demands and the management practices adopted during the reproduction phase, two sub-stages were established for calculating the amount of feed provided to the broodstock: “maintenance” and “breeding”. In the grow-out phase, the feed conversion rate (FCR) was incorporated in addition to the mentioned indicators.

**Table 5:** Nutritional welfare reference values for tilapia breeding (BR), nursery (NU), and grow-out (GO) phases and different weights^*^.

**Indicators**	**Phases**							**References**
	**Scores**	**BR**	**NU**		**GO**			
			**(** ≤ **0.5 g)**	**(0.6–5.0 g)**	**(5.1–30 g)**	**(31–150 g)**	**(151–1,000 g)**	
Feed crude protein (%)	1	30–45	40–50	32–40	35–40	28–36	28–	(32, 39, 90–99)
	2	25–29	28–39	28–31	28–34	20–27	20–27	
	3	≤ 24–≥ 46	≤ 27–≥ 51	≤ 27–≥ 41	≤ 27–≥ 41	≤ 19–≥ 37	≤ 19–≥ 37	
Amount of feed (% biomass)^**^	1	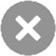	15–30	4–15	4–8	3–6	≥ 2	(30, 45, 88, 99–104)
	2	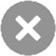	10–14	3–14	3	2	1	
	3	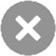	≤ 10–≥ 31	≤ 2	≤ 2	≤ 1	< 1	
Amount of feed during maintenance (% biomass)^*^	1	≥ 2	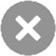	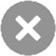	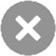	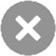	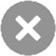	(94, 105–107)
	2	1						
	3	< 1						
Amount of feed during mating (% biomass)^*^	1	3–5						(94, 105–107)
	2	2	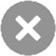	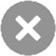	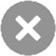	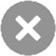	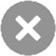	
	3	≤ 1						
Feeding frequency (times/day)	1	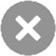	5–8	≥ 3	≥ 3	≥ 2	≥ 2	(108–111)
	2	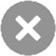	2–4	2	2	1	1	
	3	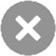	≤ 1	≤ 1	< 1	< 1	< 1	
Feeding frequency during maintenance (times/day)	1	≥ 2						(108–110)
	2	1	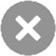	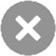	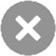	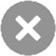	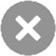	
	3	< 1						
Feeding frequency during mating (times/day)	1	> 1						(112, 113)
	2	1	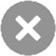	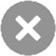	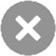	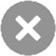	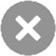	
	3	< 1						
Food distribution (% of water surface area reach)	1				≥ 75 of surface area			(23, 111)
	2				50–74 of surface area			
	3				≤ 49 of surface area			
FCR	1	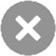	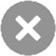	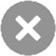	≤ 1.0	≤ 1.3	≤ 1.6	(110, 114, 115)
	2				1.1–1.6	1.4–1.7	1.7–2.0	
	3				≥ 1.7	≥ 1.8	≥ 2.1	

Not included (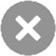) in each production phase.

^*^Fish weight adapted from Borges (116). ^**^Always, when rounding a number from one decimal place to none, if the first number after the decimal point is 5 or greater, add 1 to the number before the decimal point; if it is < 5, keep the number before the decimal point unchanged.

### 3.4. Behavioral welfare indicators for Nile tilapia

#### 3.4.1. Breeding (BR) and grow-out (GO)

Behavioral welfare indicators have been established under the management practices commonly adopted during the breeding (BR) and grow-out (GO) phases (Table 6). In both phases, the selected indicators include the effectiveness of anesthesia during invasive procedures and feeding behavior. Regarding feeding, monitoring the time required for fish to capture and entirely consume the provided food is essential. Additionally, during the grow-out phase, the swimming behavior of tilapia during harvesting was considered, along with the total time until the loss of consciousness during slaughter. This latter indicator encompasses the period from the start of the procedure until the point where the animal demonstrates a complete absence of clinical reflexes.

**Table 6:** Behavioral welfare reference values for tilapia breeding (BR) and grow-out (GO) phases.

**Stages**		**Management**	**Indicators**	**Scores**	**Reference values**	**Reference**
**BR**	**GO**					
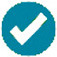	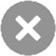	Invasive procedures (chipping, tagging, clipping)	Anesthesia–surgical stage (lack of balance and swimming; reduction of the opercular rate	1	Induction in 1–3 min; recovery in ≤ 5 min	(117, 118)
				2	Induction and or recovery in > 5 min	
				3	No induction or no recovery; death	
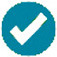	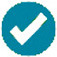	Feeding	Feed intake (minutes)	1	180–300	(23)
				2	120–179 or 301–419	
				3	≤ 119 or ≥ 420	
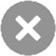	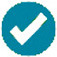	Invasive procedure (Vaccination)	Anesthesia–surgical stage (lack of balance and swimming; reduction of the opercular rate	1	Induction in 1–3 min; recovery in ≤ 5 min	(117, 118)
				2	Induction and or recovery in > 5 min	
				3	No induction or no recovery; death	
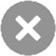	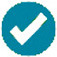	Harvest (partial or total)	Swimming behavior	1	Most fish with regular swimming and or few body parts on the surface	(23, 119)
				2	Most of the fish show restless swimming behavior, swimming in different directions and or jumping	
				3	Most fish with decreasing activity; fish trapped against the net or swimming sideways; exposure of the body to air; exhaustion	
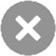	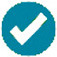	Stunning during slaughter	Reflexes^*^	1	Instantaneous loss of EQ, TGR, VER, OR	(119–121)
				2	Instantaneous loss of EQ and TGR, progressive loss of VER and OR in ≤ 30s	
				3	Progressive loss of E.Q., T.G.R., VER and OR in ≥ 31s	

^*^EQ, equilibrium; TGR, tail grab reflex; OR, opercular beating rate; VER, vestibule-ocular reflex.

#### 3.4.2. Nursery (NU) phase

Swimming behavior was selected as the sole practical and viable indicator for the visual analysis of larvae and post-larvae, establishing swimming characteristics across different phases (Table 7). The feeding behavior was also included as a welfare indicator for the post-larval stage.

**Table 7:** Behavioral welfare reference values for tilapia during the nursery (NU) phase.

**Indicators**	**Score**	**Reference values**	**References**
Larvae swimming behavior	1	Most of the sampled animals presented active swimming against the current	(70, 71, 146, 147)
	2	Most of the sampled animals presented reduced swimming activity against the current	
	3	> 10% of the sample gathered and remained immobile in the center of the container; swimming rapidly, loose equilibrium; present sideways swimming; rubbing against hard surfaces; gasping at the surface	
Post-larvae and fingerlings' swimming behavior	1	Most of the sampled animals presented active swimming against the current and swimming vertically or horizontally in the water column and with short periods at the tank bottom	(40, 70, 71, 146)
	2	Most of the sampled animals presented reduced swimming activity in the water column, or < 10% of the sample present at the tank bottom	
	3	> 10% of the sampled animals presented spiral swimming, efforts to swallow air or float on the water surface; rubbing against hard surfaces; gasping at the surface or being immobile at the tank bottom	
Post-larvae and fingerlings' feed intake (min)	1	180–300	
	2	120–179 or 301–419	(23)
	3	≤ 119 or ≥ 420	

### 3.5. Weights for calculating the partial (PWIs) and general welfare index (GWI)

The weights assigned to determine the importance of each adopted welfare indicator were established based on the number of publications identified in the Google Scholar platform. These values were obtained through a combination of general search terms (“Oreochromis niloticus” AND “aquaculture” AND the respective life phase) and specific search terms presented in Tables 8–10.

**Table 8:** Number of documents and the respective weights of the indicators, established from the general search terms (“*Oreochromis niloticus*” AND “aquaculture” AND “breeding”-larva -nursery AND the specific search terms used in Google Scholar in July 2023).

**Freedom**	**Indicator**	**Specific search terms**	**Number of documents (*n*)**	**Weight [ln(*n*)]**
Environmental	Alkalinity	“alkalinity”	1.510	7
	Aquatic predators and other interspecific inhabitants	“aquatic predators” OR “interspecific inhabitants”	43	4
	Dissolved oxygen	“dissolved oxygen”	6.780	9
	Hapas net cleaning	“hapa” AND “clean”	76	4
	Nitrite	“nitrite”	2.560	8
	Non-ionized ammonia	“ammonia”	5.250	9
	pH	“pH”	13.500	10
	Photoperiod	“photoperiod”	3.460	8
	Sex ratio (male: female)	“sex ratio”	2.550	8
	Temperature	“temperature”	15.500	10
	Terrestrial predators	“terrestrial” AND “predator”	627	6
	Transparency	“transparency”	976	7
Health	Breeding control	“breeding control”	10	2
	Conditioning/breeding interval (days)	“conditioning” OR “breeding interval”	729	7
	Eggs–macroscopical aspects	“eggs” AND “macroscopic”	240	5
	Eyes	“eyes”	674	7
	Fins	“fins”	2.480	8
	Gills	“gills”	4.000	8
	Invasive procedures	“chipping” OR “tagging” OR “clipping.”	847	7
	Jaws/lips	“jaw” OR “lips”	857	7
	Mortality (%)	“mortality”	8.020	9
	Operculum	“operculum”	573	6
	Sexual maturation	“maturation”	5.510	9
	Skin	“skin”	5.090	9
	Spine	“spine”	657	6
Nutritional	Amount of feed	“amount of feed”	910	7
	Feed Crude Protein	“crude protein”	4.700	8
	Feeding frequency	“feeding frequency”	834	7
	Food distribution	“food distribution”	91	5
Behavioral	Anesthesia–surgical stage	“anesthesia” OR “anesthesia”	486	6
	Feed intake	“feed intake”	3.290	8
	Swimming behavior	“swimming behavior” OR “swimming behavior”	432	6

**Table 9:** Number of documents and the respective weights of the indicators, established from the general search terms (“*Oreochromis niloticus*” AND “aquaculture” AND nursery” OR “larviculture” – breeding AND the specific search terms used in Google Scholar in July 2023 + specific search terms).

**Freedom**	**Indicator**	**Specific search terms**	**Number of documents (*n*)**	**Weight [ln(*n*)]**
Environmental	Alkalinity	“alkalinity”	932	7
	Aquatic predators and other interspecific inhabitants	“aquatic predators” OR “interspecific inhabitants”	9	2
	Dissolved oxygen	“dissolved oxygen”	2.810	8
	Nitrite	“nitrite”	1.550	7
	Non-ionized ammonia	“ammonia”	2.620	8
	pH	“ph”	4.850	8
	Photoperiod	“photoperiod”	979	7
	Temperature	“temperature”	4.660	8
Health	Emaciation state	“emaciation”	35	4
	Eyes	“eyes”	273	6
	Fins	“fins”	569	6
	Hatching rate	“hatching”	1.010	7
	Jaws/lips/head	“jaw” OR “lips” OR “head”	1.350	7
	Mortality (%)	“mortality”	2.780	8
	Operculum	“operculum”	134	5
	Skin	“skin”	1.230	7
	Spine	“spine”	134	5
	Tail	“tail”	523	6
	Yolk sac	“yolk sac”	364	6
Nutritional	Amount of feed	“amount of feed”	550	6
	Feed crude protein	“crude protein”	2.090	8
	Feeding frequency	“feeding frequency”	62	4
	Food distribution	“food distribution”	37	4
Behavioral	Feed intake	“feed intake”	13,50	7
	Swimming behavior	“swimming behavior”	918	7

**Table 10:** Number of documents and the respective weights of the indicators established from the general search terms (“*Oreochromis niloticus*” AND “aquaculture” AND “farming” AND “pond” AND the specific search terms used in Google Scholar in July 2023).

**Freedom**	**Indicator**	**Specific search terms**	**Number of documents (*n*)**	**Weight [ln(*n*)]**
Environmental	Alkalinity	“alkalinity”	2.850	8
	Aquatic predators and other interspecific inhabitants	“aquatic predators” OR “interspecific inhabitants”	44	4
	Dissolved oxygen	“dissolved oxygen”	8.500	9
	Nitrite	“nitrite”	4.310	8
	Non-ionized ammonia	“ammonia”	7.380	9
	pH	“pH”	12.500	9
	Temperature	“temperature”	12.900	9
	Terrestrial predators	“terrestrial” AND “predator”	533	6
	Transparency	“transparency”	202	5
Health	Eyes	“eyes”	196	5
	Fins	“fins”	255	6
	Gills	“gills”	370	6
	Invasive procedures	“chipping” OR “tagging”	505	6
	Jaws/lips	“jaw” OR “lips”	467	6
	Mortality (%)	“mortality”	7.740	9
	Operculum	“operculum”	449	6
	Skin	“skin”	3.830	8
	Spine	“spine”	355	6
Nutritional	Amount of feed	“amount of feed”	1.710	7
	Feed conversion ratio (FCR.)	“F.C.R.”	4.550	8
	Feed crude protein	“crude protein”	107	5
	Feeding frequency	“feeding frequency”	1.260	7
	Food distribution	“food distribution”	199	5
Behavioral	Feed intake	“feed intake”	87	4
	Harvest (partial or total)—swimming behavior	“swimming behavior” OR “swimming behavior”	330	6
	Invasive procedure (vaccination)—Anesthesia—surgical stage	“vaccination”	1.060	7
	Stunning during slaughter—reflexes	“stunning”	129	5

**Figure 3:**
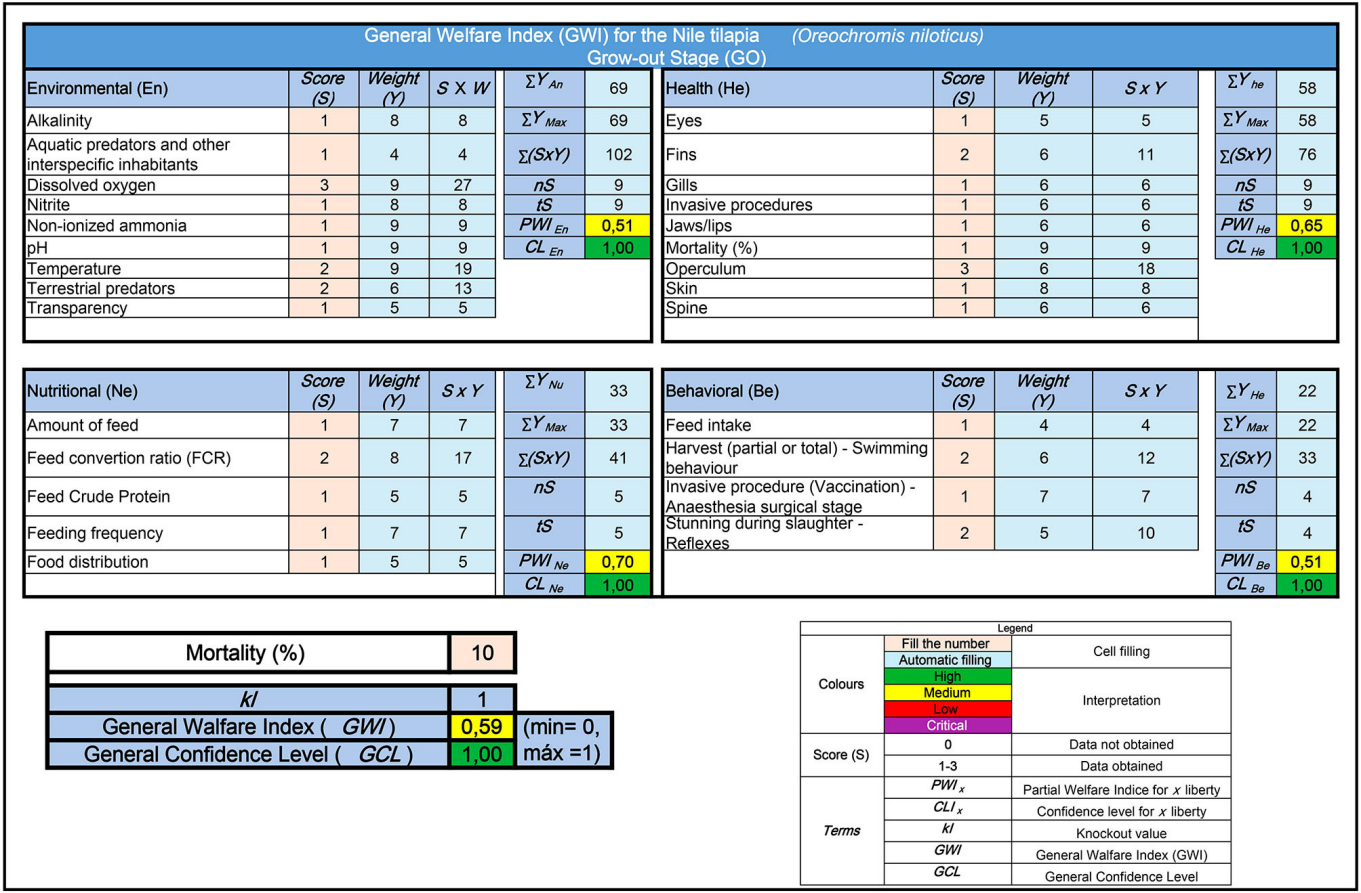
Example of calculating partial welfare indices for tilapia during the grow-out phase in land-based ponds and the overall welfare index using a calculation model developed in the Microsoft Excel application. In this hypothetical case, the model indicates a moderate level of welfare and a high level of confidence concerning this result.

Figure 3 illustrates the application of the welfare protocol for *O. niloticus* during the grow-out phase. The data were simulated using a Microsoft Excel spreadsheet and are derived from a hypothetical but commonly observed scenario in commercial tilapia farming. Based on the example, partial welfare indices related to the environment (PWI_En_), health (PWI_He_), nutrition (PWI_Nu_), and behavior (PWI_Be_) of the fish are calculated. In the analyzed, the first three indices indicate a moderate level of welfare for the cultivated fish. However, the behavioral index suggests a low level of welfare due to improper management practices during harvesting and slaughter. The simulated scenario calculated the general welfare index (GWI) at 0.59, considered moderate. The confidence level in this result was the highest, as all indicators were analyzed. Examples of these index calculations during the breeding and nursery phases are included in Supplementary Figures 1, 2.

## 4. Discussion

The current paradigm regarding the welfare of farmed fish suggests that the evaluation parameters used should be specific to each species, considering the animals' developmental stage and the system in which they are raised. These parameters should also encompass indicators that address the fish's physical, nutritional, environmental, and behavioral aspects (13, 23, 25, 26, 87, 119, 122). However, the understanding and investigation of the psychological dimensions, although constituting one of the animal freedoms, represent a field of scientific knowledge still in its early and nascent stages, especially when compared to the progress achieved regarding animals involved in terrestrial agriculture. Notably, the impacts of domestication on the welfare of farmed fish are more complex to analyse than those faced in welfare studies of land animals that serve as human food sources (123). This is because fish have significantly different genetic, physiological, and behavioral characteristics compared to land animals, as well as experiencing a completely different sensory universe (124, 125). Thus, developing empathy for fish and understanding their needs entails a series of challenges to be evaluated in the field, which makes it impractical to include them in operational welfare protocols for tilapia.

The welfare of any organism is a dynamic state (126, 127), and the systems used to monitor it should be flexible enough to adapt to changes in its welfare state (17). There is also an understanding of the need for protocols to consider the interaction between different indicators (119, 122, 128), providing relevant information about the overall quality of life of the animals (129, 130). In this context, despite the recent trend of an increasing number of physiological or molecular parameters being tested and recommended and despite the effectiveness of these indicators in laboratory conditions (131–135), the proposed welfare indicators should apply to the practical requirements and routines of commercial fish breeding, larviculture, and grow-out operations (13, 17, 136).

Thus, good operational welfare indicators for farmed fish can be defined as those that address biologically relevant aspects, are easy to use, preferably non-invasive and low-cost (130, 137); reliable, comparable, suitable for aquaculture practices, and appropriate for specific systems or routines (16). They should identify welfare problems and risks to animal welfare and serve as a basis for technical decision-making by producers (138), enabling timely corrections. In contrast, it is highly unlikely that expensive, complex, unreliable, or time-consuming tools or techniques will be adopted and incorporated by the aquaculture industry (139).

### 4.1. Changes in the welfare protocol developed for the grow-out phase of tilapia

When we originally proposed the method and indices that our group has already applied to assess the degree of welfare in grass carp and white-leg shrimp (25, 26), we emphasized that the indicators, their reference values, scores, and weights would need to be periodically reviewed and updated as scientific knowledge advances on the subject. In this article, we put this concept into practice by applying our metrics to assess the welfare of tilapia not only during the grow-out phase but throughout all life stages, whilst also revising and advancing the knowledge generated previously. The indicators now applied to tilapia in the grow-out phase had already been tested and validated by our group under field conditions (23). In this article, in addition to revising and updating the indicators and reference values for this cultivation phase, we simplified the evaluation structure by reducing the number of scores for each indicator from four to three. This approach improves field evaluation, making it more objective and dynamic than protocols with higher scores (87, 140). This reduction in scores regarding changes affecting eyes, gills, and fins, for example, which often indicate pain and significant diseases, reduces subjectivity in interpreting moderate lesions, thus enhancing evaluation accuracy. However, the blood glucose indicator was removed from the current protocol due to the invasiveness of the method. Additionally, it should be considered that the blood sampling procedure itself can alter the parameters, causing acute stress to the animals.

Regarding environmental indicators such as temperature, pH, and dissolved oxygen, it is necessary to consider that these parameters naturally vary throughout the day in cultivation ponds. However, the animals can adapt (141) as long as the changes occur within tolerable limits for the species. To avoid conflicts between different values considered acceptable in the literature, we adjusted the new scores to reflect these possible changes in water quality in tilapia cultivation ponds.

The stocking density indicator was removed from the current protocol. Although it is evident that stocking density directly influences the degree of welfare and fish health (142–144), establishing fixed values for this parameter is highly subjective. Defining the ideal density regarding welfare depends significantly on the characteristics of the fish, environmental and nutritional resources provided to them, management practices, fish size, genetic characteristics, and other factors (145).

We also found several operational and conceptual difficulties regarding the shading indicator proposed in the previous protocol. Recent studies present conflicting data that do not accurately reflect the reality of pond cultures, mainly due to inadequate consideration of light intensity and its impact on water quality (148, 149). Initially, we proposed a uniform percentage of shading over the surface of the net pen or pond, typically achieved by using protective and shading screens. However, ponds often have localized shade caused by trees or topographic features in their surrounding areas. This led to misinterpretations during the application of the protocol since localized shading is detrimental to the welfare of tilapia (150). Therefore, we chose to exclude this indicator from the current protocol.

In the original protocol for tilapia grow-out, we established crude protein content (CP), feed conversion ratio (FCR), condition factor (K), and feeding behavior as nutritional indicators. However, after reviewing the data from applying the protocol in various countries (Pedrazzani, unpublished data), we found significant genetic variability amongst cultivated *O. niloticus* strains, which led to morphological variations in the fish. Sometimes the strains were naturally broader than long, and *vice versa*, which affected the value of the condition factor (K) without any relation to fish welfare. Therefore, adjusting the formula for each population or strain of the same species proved impractical for standardization.

The nutritional freedom indicator has now been adjusted to calculate welfare indices by category. Thus, to facilitate tracking the feeding history on farms, we standardized four indicators for all cultivation phases: feed quantity, protein content, feeding frequency, and feed distribution within the pond. Due to the difficulty of capturing and weighing fish in early cultivation, FCR monitoring was suggested only for the grow-out phase.

The behavioral indicators during feeding, harvesting, and slaughter were kept the same as in the original protocol, but their scores were readjusted, aiming to reduce subjectivity during the evaluation, as previously discussed regarding animal suffering associated with health freedom.

An essential conceptual aspect concerns fish slaughter, which we associate with the grow-out phase. Although the industrial-scale slaughter of tilapia is already a reality (151, 152), globally, this practice remains an exception rather than the rule. Transporting water and live tilapia to the processing plants is costly and requires complex logistics and appropriate slaughter facilities. In most cases, fish are sold alive in markets or slaughtered on-site at the farms where they are cultivated (29, 39, 57, 152–154). Under these circumstances, it is common for animals to be slaughtered without prior stunning, often asphyxiated in the air or ice (155–157). Thus, we believe that a more practical way to improve fish welfare is to consider slaughter as part of the grow-out process. However, considering the expansion of international tilapia trade, the evolution of industrial aquaculture, and the increasing interest of consumers in the quality of the product and the welfare of farmed fish, it is plausible to project that slaughter will soon become a genuinely autonomous stage in the tilapia production cycle.

### 4.2. First protocol of tilapia welfare for the reproduction and nursery stages

The sanitary indices employed during the grow-out phase were maintained with those developed for adult fish and further expanded. The additions were intended to integrate essential management practices commonly used in commercial tilapia reproduction and larviculture facilities. Indicators such as the stage of sexual maturation, the time interval adopted for the recovery/conditioning of breeders between reproductive cycles, and the assessment of the adoption or not of methods or mechanisms of control to avoid inbreeding amongst breeder batches were included. In addition to these, other less intuitive indicators were proposed, such as, for instance, the maximum intervals adopted for cleaning the hapas where the breeders are kept during reproduction—an essential factor to prevent the obstruction of the screens since this hinders the renewal of water and compromises the health of the breeders and can cause not only a reduction of zootechnical indices but also a decrease in immunity and the emergence of diseases (30, 57, 158). The proportion between males and females used during reproduction was another indicator included in the protocol since it interferes with population dynamics and the degree of aggressiveness of the males during the mating phase (30).

For the behavioral assessment of the breeders, we emphasize the recommendation for using anesthesia during any invasive procedures. We kept the “feeding behavior” indicator, although it is relevant to note that female tilapias reduce their food intake during the mating and spawning periods by incubating the eggs in the mouth, whilst males can increase their feed intake in the same period (159). This protocol included the photoperiod due to its central role in the natural induction of sexual maturation and in defining reproductive rates (160, 161).

Concerning the welfare of eggs, larvae, and post-larvae, considering that this is still a controversial topic, that it is in its initial stages of unveiling scientific knowledge, and that there is a tremendous natural vulnerability of larvae and post-larvae to handling, which requires even greater caution and rigor; we advocate for the implementation of the protocol proposed in this phase of tilapia cultivation. However, at the same time, we suggest that the suitability and necessity of its application be determined on a case-by-case basis, considering the overall conditions of the batch and the local structural and operational capacity to assess the proposed indicators.

The welfare of eggs and larvae involves the parents' nutritional, social, and environmental experiences during their development (162). The environment likely influences the epigenetic pattern of gametes, embryos, or adult organisms (163). During gametogenesis, the DNA is reprogrammed, and this information will be transmitted to the offspring, resulting in transgenerational effects that directly impact the quantity, viability, social status, neurogenesis, and adaptation of future generations (162). Sneddon et al. (1) highlight that fish larvae have various brain structures that process emotions and learning, although they are not identical to the human brain. Lopez et al. (164) demonstrated that zebrafish larvae at 5 days post-fertilization (5 daf) respond to harmful and potentially painful stimulation caused by environmental acidification, exhibiting similar behaviors to adult fish and reducing their activities. This response was alleviated by analgesic drugs such as lidocaine and morphine. Furthermore, different larval rearing protocols can have a significant impact on larval size and mass, survival rates, and the sex ratio of larvae (165). Therefore, it is necessary to consider that welfare should be understood as continuous and intergenerational, as there is a direct link between offspring adaptation and the resources provided by parents (162, 166). On the other hand, the quality of the eggs and larvae will also significantly impact the welfare and health of tilapias throughout their lives (167).

Another argument to be considered is that, despite the legislation and regulatory frameworks of the vast majority of countries still not protecting fish larvae, we must consider the scientific arguments linked to the presence of sentience in larvae and fry (1, 162, 164). In this sense, we advocate the application of the proposed protocol based on the precautionary principle, which establishes that when evidence of sentience is inconclusive, we should “give the benefit of the doubt” to the animal or “err on the side of caution” (168). Thus, we understand that the theme “welfare in the early stages of fish life” has relevance to be applied in current aquaculture but, mainly, that it will have a significant impact on the aquaculture that will be practiced in the coming years, possibly under a scenario of regulatory restrictions and rigorous governance practices (169). Therefore, be it for biological, ethical, moral, or commercial reasons, and even recognizing the fragility of the current stage of knowledge on the subject and the need for subsequent discussions on the effectiveness of the application of welfare protocols for the larval and post-larval stages of *O. niloticus*, we understand it to be recommendable and, at the same time, almost “inevitable” that the early stages of life be included in animal welfare assessment protocols in fish farming.

We tested and validated their operational feasibility in the laboratory to assess the indicators proposed here for the early stages of tilapia life. The experiments carried out made it possible to identify the most suitable indicators and, at the same time, exclude those that did not meet the established prerequisites.

Some health indicators were not incorporated into the larval protocols, as structures such as fins and gills are still in development during the ontogenetic processes that occur during the nursery phase, making their visual assessment difficult. For post-larvae and fry, it is relevant to consider the “degree of emaciation” as an indirect indicator of feeding effectiveness. The “yolk sac” indicator was included in the assessment of larvae, as organisms at this stage of life still have endogenous energy reserves and, therefore, do not show apparent signs of emaciation (76). In all life stages, we kept the mortality rate as an indicator of welfare, as most fish deaths in captivity are likely preceded or accompanied by suffering. Thus, long-term mortality rates may serve as indicators of the degree of retrospective welfare and signal possible future impacts on the success rates of the enterprise (170).

In the analysis of tilapia larvae and post-larvae, we identified a significant number of articles focussed solely on swimming behavior and the changes commonly related to water contamination or the occurrence of diseases. Therefore, we included only this behavioral indicator for the larval stage once they have endogenous feeding. We also excluded some indicators from this stage, as larvae are produced in laboratories, rendering indicators such as the presence of “terrestrial predators” or “water transparency” irrelevant, for instance.

### 4.3. Partial and general welfare index (PWIs) and general welfare index (GWI)

Animal welfare should not be linked to cultural differences or subjective criteria but to the species' biology (171). Therefore, quantitative animal welfare assessment is essential for promoting humane and responsible management practices in the animal production industry (172, 173). Moreover, using quantitative and standardized approaches in welfare measurement allows producers to tangibly demonstrate their commitment to animal welfare, which helps build consumer trust and generate new market opportunities (174, 175).

The metrics proposed in this article aim, pioneeringly, to provide a holistic and quantifiable assessment of the welfare of Nile tilapia throughout all stages of its captive life cycle. These metrics were established based on indicators that were simultaneously simple, understandable, and already part of the routine production of the species on a commercial scale. To achieve this, we used indicators representing the nutritional, behavioral, health, and critical environmental conditions to which tilapia are exposed throughout their production process.

Furthermore, this study's partial (PWI) and overall (GWI) welfare indices offer an objective animal welfare assessment. They are based on data and scientifically supported metrics rather than opinions or subjective factors, providing excellent reliability and accuracy. The proposed indices can provide producers with a valuable tool for retrospective and prospective analyses within the same production cycle, enabling informed strategic decisions for the welfare of farmed fish and the profitability and efficiency of their businesses.

There is already recognition within the scientific community that a single score simplifies data interpretation and constitutes a valuable tool for researchers, producers, certifying bodies, and regulatory agencies (87, 122). This characteristic allows the proposed indices to establish a solid foundation for developing animal welfare regulations and guidelines, enabling authorities to define clear and measurable standards to ensure ethical and humane treatment in tilapia farming operations, one of the most critical species in global aquaculture (18).

In this study, PWIs and GWIs follow the same conceptual and mathematical logic applied and extensively discussed concerning grass carp (25) and white-leg shrimp (26). However, the indicators and their respective reference values, scores, and weights are specific to *O. niloticus*, covering aspects of breeding, larviculture, fingerling rearing, and the grow-out phase—in this case, in earthen ponds. Like in previous studies, the weights assigned to each indicator were identified using Google Scholar. These weights ranged from 2 to 10 and were determined based on the number of scientific documents related to each indicator. It should be noted, however, that despite being practical, this method has limitations. For example, the number of publications on a specific topic may not reflect its relevance in a practical context; it may underestimate the importance of less-researched welfare indicators; it can be influenced by the availability of funding for research in specific areas, which may not reflect the importance of those areas for animal welfare; there may also be variations due to the language used in search terms and differences in the consulted databases. Despite these limitations, the approach is robust, standardisable, and encompasses several advantages, including the comprehensiveness and objectivity of evaluating welfare in tilapia farming, recognizing differences that each indicator presents in fish welfare, and the ability to update and refine as new research is published. Subsequent analysis can explore alternative methods for assigning weights to welfare indicators and further examine how the relative importance of these indicators may vary across different life stages.

## 5. Conclusion

In this study, we have proposed a comprehensive and quantitative approach to assess the welfare of Nile tilapia throughout their life cycle (eggs, larvae, post-larvae, juveniles, and adults) and all cultivation phases (breeding, nursery, and grow-out) in captivity. This approach has generated a valuable and standardized tool for aquaculturists to monitor and improve their production systems, with the potential to enhance the welfare of *O. niloticus* in aquaculture significantly.

The proposed methods will allow for a comprehensive, precise, and tailored analysis of welfare throughout the entire life cycle and all stages of tilapia farming. The developed quantitative indices will enable a standardized comparison of animal welfare amongst different enterprises, locations, and periods, serving as relevant tools to evaluate the effectiveness of other management practices and identify areas that need improvement. This approach can potentially enhance farming practices and promote the welfare of tilapia whilst providing a valuable tool for advancing more sustainable and ethical aquaculture practices.

## Data availability statement

The raw data supporting the conclusions of this article will be made available by the authors, without undue reservation.

## Ethics statement

The animal study was approved by Animal Use Ethics Committee of the Agricultural Sciences Campus (CEUA) of the University of Paraná under protocol 021/2023. The study was conducted in accordance with the local legislation and institutional requirements.

## Author contributions

AP: Conceptualization, Data curation, Formal analysis, Investigation, Validation, Writing—original draft, Writing—review and editing. NC: Investigation, Methodology, Resources, Validation, Writing—original draft. MQ: Project administration, Supervision, Writing—review and editing. CT: Investigation, Validation. VB: Validation. AO: Conceptualization, Methodology, Investigation, Formal analysis, Writing—original draft, Writing—review and editing, Supervision, Project administration.

## References

[1] Sneddon LY Lopez-Luna J Wolfenden DCC Leach MC Valentim AM Steenbergen PJ. Fish sentience denial: muddying the waters. Anim Sentience. (2018) 115:1–12. 10.51291/2377-7478.1317

[2] Chandroo KP Duncan IJH. Moccia RD. Can fish suffer? Perspectives on sentience, pain, fear and stress. Appl Anim Behav Sci. (2004) 86:225–50. 10.1016/j.applanim.2004.02.004

[3] Ashley PJ. Fish welfare: Current issues in aquaculture. Appl Anim Behav Sci. (2007) 104:199–235. 10.1016/j.applanim.2006.09.001

[4] Braithwaite V. Do Fish Feel Pain? Oxford: Oxford University Press (2010) 208 p.

[5] UK Public General Acts. Animal Welfare (Sentience) Act 2022 Chapter 22. (2022). Available online at: https://www.legislation.gov.uk/ukpga/2022/22/contents (accessed June 10, 2023).

[6] Gismervik K Tørud B Kristiansen TS Osmundsen T Størkersen KV Medaas C. Comparison of Norwegian health and welfare regulatory frameworks in salmon and chicken production. Rev Aquac. (2020) 12:2396–410. 10.1111/raq.12440

[7] Giménez-Candela MM Saraiva JL Bauer H. The legal protection of farmed fish in Europe – analysing the range of EU legislation and the impact of international animal welfare standards for the fishes in European aquaculture. Derecho Animal Forum Animal Law Stud. (2020) 11:65–119. 10.5565/rev/da.460

[8] Seibel H Weirup L Schulz C. Fish welfare – between regulations, scientific facts and human perception. Food Ethics. (2020) 5:1–11. 10.1007/s41055-019-00063-3

[9] Poli BM. Farmed fish welfare-suffering assessment and impact on product quality. Ital J Anim Sci. (2009) 8:139–60. 10.4081/ijas.2009.s1.139

[10] Newton R Zhang W Xian Z McAdam B Little DC. Intensification, regulation and diversification: the changing face of inland aquaculture in China. Ambio. (2021) 50:1739–56. 10.1007/s13280-021-01503-3 33675016 PMC7935007

[11] Maesano G Di Vita G Chinnici G Pappalardo G D'Amico M. The role of credence attributes in consumer choices of sustainable fish products: a review. Sustainability. (2020) 12:10008. 10.3390/su122310008

[12] Kaimakoudi E. Policy initiatives towards enhancing consumer knowledge and tackling consumer confusion in aquaculture sector. Aquac Int. (2023) 2023:1–9. 10.1007/s10499-023-01143-2

[13] Barreto MO Rey Planellas S Yang Y Phillips C Descovich K. Emerging indicators of fish welfare in aquaculture. Rev Aquac. (2022) 14:343–61. 10.1111/raq.12601

[14] Toni M Manciocco A Angiulli E Alleva E Cioni C Malavasi S. Review: assessing fish welfare in research and aquaculture, with a focus on European directives. Animal. (2019) 13:161–70. 10.1017/S1751731118000940 29717679

[15] Størkersen KV Osmundsen TC Stien LH Medaas C Lien ME Tørud B. Fish protection during fish production. Organizational conditions for fish welfare. Mar Policy. (2021) 129:104530. 10.1016/j.marpol.2021.104530

[16] Nilsson J Stien LH Iversen MH Kristiansen TS Torgersen T Oppedal F. Part A. Knowledge and theoretical background. In:Noble C Gismervik K Iversen MH Kolarevic J Nilsson J Stien LH et al., editors. Welfare Indicators for Farmed Atlantic Salmon: Tools for Assessing Fish Welfare. Tromsø: Nofima (2018). p. 351.

[17] Segner H Reiser S Ruane N Rösch R Steinhagen D Vehanen T. Welfare of Fishes in Aquaculture. Budapest: FAO (2019). 18 p.

[18] FAO. The State of World Fisheries and Aquaculture 2022. Towards Blue Transformation. Rome, Italy: FAO (2022). 266 p.

[19] El-Sayed A-FM Fitzsimmons K. From Africa to the world—the journey of Nile tilapia. Rev Aquac. (2023) 15:6–21. 10.1111/raq.12738

[20] Rodriguez-Barreto D Rey O Uren-Webster TM Castaldo G Consuegra S Garcia de. Leaniz C. Transcriptomic response to aquaculture intensification in Nile tilapia. Evol Appl. (2019) 12:1757–71. 10.1111/eva.12830 31548855 PMC6752142

[21] Huntingford F Kadri S. Defining, assessing and promoting the welfare of farmed fish. Rev Sci Tech. (2014) 33:233–44. 10.20506/rst.33.1.2286 25000796

[22] Daskalova A. Farmed fish welfare: stress, post-mortem muscle metabolism, and stress-related meat quality changes. Int Aquat Res. (2019) 11:113–24. 10.1007/s40071-019-0230-0

[23] Pedrazzani AS Quintiliano MH Bolfe F Sans ECdO Molento CFM. Tilapia on-farm welfare assessment protocol for semi-intensive production systems. Front Vet Sci. (2020) 7:1–16. 10.3389/fvets.2020.606388 33324705 PMC7723968

[24] FAWC. Prevention of Cruelty to Animals Act. (1979). Available online at: https://www.gov.uk/government/groups/farm-animal-welfare-committee-fawc (accessed March 15, 2023).

[25] Pedrazzani AS Tavares CSP Quintiliano M Cozer N Ostrensky A. New indices for the diagnosis of fish welfare and their application to the grass carp (*Ctenopharyngodon idella*) reared in earthen ponds. Aquac Res. (2022) 53:5825–45. 10.1111/are.16105

[26] Pedrazzani AS Cozer N Quintiliano MH Tavares CPdS Silva UAT Ostrensky A. Non-invasive methods for assessing the welfare of farmed white-leg shrimp (Penaeus vannamei). Animals. (2023) 13:807. 10.3390/ani13050807 36899664 PMC10000178

[27] Whittemore R Knafl K. The integrative review: updated methodology. J Adv Nurs. (2005) 52:546–53. 10.1111/j.1365-2648.2005.03621.x 16268861

[28] Ross LG Ross B. Anaesthetic and sedative techniques for aquatic animals. Ross LG, Ross B, editors. Nova Jersey. Hoboken, NJ: John Wiley & Sons (2009). 222 p.

[29] Nandlal S Pickering T. Tilapia Fish Farming in Pacific Island Countries. Noumea, New Caledonia: Secretariat of the Pacific Community (2004). 190–203 p.

[30] El-Sayed AFM. Tilapia Culture: Second Edition. Massachusetts: Elsevier (2019). 348 p.

[31] López-Olmeda JF Noble C Sánchez-Vázquez FJ. Does feeding time affect fish welfare? Fish Physiol Biochem. (2012) 38:143–52. 10.1007/s10695-011-9523-y 21671025

[32] El-Naggar G El Nady M Kamar M Al-Kobabay A. Effect of photoperiod, dietary protein and temperature on reproduction in Nile tilapia (*Oreochromis niloticus*). Tilapia Culture in the 21st Century. In: *Proceedings from the Fifth International Symposium on Tilapia Aquaculture*. Rio de Janeiro, Brazil: American Tilapia (2000). p. 352–8.

[33] FAO. The State of World Fisheries and Aquaculture. (2009). Available online at: https://www.fao.org/fishery/en (accessed May 24, 2023).

[34] Bhujel RC A. review of strategies for the management of Nile tilapia (*Oreochromis niloticus*) broodfish in seed production systems, especially hapa-based systems. Aquac. (2000) 181:37–59. 10.1016/S0044-8486(99)00217-3

[35] Alam SMA Sarkar MSI Miah MMA Rashid H. Management strategies for nile tilapia (*Oreochromis niloticus*) hatchery in the face of climate change induced rising temperature. Aquac Stud. (2021) 21:55–62. 10.4194/2618-6381-V21_2_02

[36] Kubitza F. Tilápias: Qualidade da água, sistemas de cultivo, planejamemto da produção, manejo nutricional e alimentar e sanidade. Parte II. In:Filho JC, editor. Tilapias. Rio de Janeiro, Brazil: Revista Panorama da Aquicultura (2015). p. 7.

[37] SEBRAE. Guia técnico para empreender na criação de tilápias em tanques-rede. 1 ed. Brasília, DF: Serviço Brasileiro de Apoio às Micro e Pequenas Empresas–SEBRAE (2016). 84 p.

[38] SENAR. Piscicultura: manejo da produção de peixes em viveiros. 1 ed. Brasília: Serviço Nacional de Aprendizagem Rural (2017). 124 p.

[39] El-Sayed AFM. Tilapia Culture. Massachusetts, USA: Cabi Publishing (2006). 277 p.

[40] Benli AÇK Koksal G. The acute toxicity of ammonia on Tilapia (Oreochromis niloticus L) larvae and fingerlings. Turk J Vet Anim Sci. (2005) 29:339–44.

[41] Costa FFB. Efeito agudo e subcrônico da amônia sobre a Tilápia do Nilo. (dissertation/master's thesis). Belo Horizonte, Brazil: Universidade Federal de Minas Gerais (2018).

[42] El-Shafai SA El-Gohary FA Nasr FA Van Der Steen NP Gijzen HJ. Chronic ammonia toxicity to duckweed-fed tilapia (*Oreochromis niloticus*). Aquac. (2004) 232:117–27. 10.1016/S0044-8486(03)00516-7

[43] Atwood HL Fontenot QC Tomasso JR Isely JJ. Toxicity of nitrite to nile tilapia: effect of fish size and environmental chloride. N Am J Aquac. (2001) 63:49–51. 10.1577/1548-8454(2001)063 < 0049:tontnt>2.0.co;2

[44] Ferreira GdS Marcondes Maciel L Dalmass MV Tiepo MG. Tilápia-do- Nilo: Criação e cultivo em viveiros no estado do Paraná. Curitiba, PR: GIA (2015). 290 p.

[45] Silva BC Massago H Marchiori NdC. Monocultivo de tilápia em viveiros escavados em Santa Catarina. Florianópolis, SC: Empresa de Pesquisa Agropecuária e Extensão Rural de Santa Catarina (Epagri) (2019). 126 p.

[46] Silva MJdS. Efeito agudo da amônia e do nitrito em tilápias Oreochromis niloticus mantidas em baixa salinidade [dissertation/master's thesis]. Belo Horizonte, Brazil: Universidade Federal de Minas Gerais (2013).

[47] Wang Y Zhang W Li W Xu Z. Acute toxicity of nitrite on tilapia (*Oreochromis niloticus*) at different external chloride concentrations. Fish Physiol Biochem. (2006) 32:49–54. 10.1007/S10695-005-5744-2 20035478

[48] Boyd CE Tucker CS. Pond Aquaculture Water Quality Management. Massachusetts, USA: Kluwer Academic Publishers (1998). 700 p.

[49] Kubitza F Kubitza LMM. Tilápias: qualidade da água, sistemas de cultivo, planejamento da produção, manejo nutricional e alimentar e sanidade—parte I. In:Filho JC, editor. Tilapia: Um bom planejamento gera alta rentabilidade. Rio de Janeiro, Brazil: Revista Panorama da Aquicultura (2000). p. 45–7.

[50] Rojas N Mainardes-Pinto C Rocha O Silva A. Larviculture of *Oreochromis niloticus* Linnaeus, 1758 (Perciformes, Cichlidae) in ponds with different levels of water alkalinity. Acta Limnol Bras. (2004) 16:341–9.

[51] Cavalcante DdH. Relação dureza/alcalinidade da água e seus efeitos sobre a qualidade da água, do solo e desempenho zootécnico de juvenis de tilápia do Nilo, Oreochromis niloticus, mantidos em condições laboratoriais (dissertation/master's thesis). Ceará, Brazil: Universidade Federal do Ceará (2012).

[52] Baroiller J-F Desprez D Carteret Y Tacon P Borel F Hoareau M-C. Influence of environmental and social factors on the reproductive efficiency in three tilapia species, *Oreochromis niloticus*, O. aureus, and the red tilapia (Red Florida strain). In:Baroiller J-F, editor. Proceedings of the Fourth International Symposium on Tilapia in Aquaculture. Actes du 4ème congrès international sur tilapia en aquaculture. Orlando, US: Northeast Regional Agricultural Engineering Service (1997). p. 808.

[53] Biswas AK Morita T Yoshizaki G Maita M Takeuchi T. Control of reproduction in Nile tilapia Oreochromis niloticus (L) by photoperiod manipulation. Aquac. (2005) 243:229–39. 10.1016/J.AQUACULTURE.2004.10.008

[54] El-Sayed AFM Kawanna M. Effects of photoperiod on growth and spawning efficiency of Nile tilapia (Oreochromis niloticus L) broodstock in a recycling system. Aquac Res. (2007) 38:1242–7. 10.1111/J.1365-2109.2007.01690.X

[55] Ridha MT Cruz EM. Effect of light intensity and photoperiod on Nile tilapia *Oreochromis niloticus* L. seed production. Aquac Res. (2000) 31:609–17. 10.1046/J.1365-2109.2000.00481.X

[56] Abdel-Satar AM Al-Khabbas MH Alahmad WR Yousef WM Alsomadi RH Iqbal T. Quality assessment of groundwater and agricultural soil in Hail region, Saudi Arabia. Egypt J Aquat Res. (2017) 43:55–64. 10.1016/j.ejar.2016.12.00426329266

[57] Bhujel R. A manual for Tilapia business management. Bhujel RC, editor. Wallingford. Leesburg: Cabi (2014). 216 p.

[58] Akian DD Quenum CL Koua DNZ Houra JAK Clota F Bégout ML. Comparative study of larvae production by the Nile tilapia (*Oreochromis niloticus*, Linné, 1758) Bouaké strain between earthen ponds and hapas. Aquac Res. (2022) 53:6049–55. 10.1111/are.16075

[59] Huisman EA Zonneveld N Bouwmans AH. Aquacultural Research in Asia: Management Techniques and Nutrition: Proceedings of the Asian Seminar on Aquaculture. Wageningen, Netherlands: Pudoc (1989). 271 p.

[60] Noga EJ. Fish disease: Diagnosis and Treatment. Second ed. North Carolina, US: Wiley-Blackwell (2010). 544 p.

[61] Smith SA. Fish Diseases and Medicine. First ed. Florida, US: CRC Press (2019). 412 p.

[62] Eiras JC Segner H Wahli T Kapoor BG. Fish Diseases (2 Vols.). First ed. Einfield, UK: Taylor and Francis Inc (2008). 1338 p.

[63] Tave D Jo JY Kim DS. Gross abnormalities in tilapia. Fish Aquat Sci. (2011) 14:148–60. 10.5657/FAS.2011.0148

[64] Handwerker TS Tave D. Semioperculum: a nonheritable deformity in mozambique tilapia. J Aquat Anim Health. (1994) 6:85–8. 10.1577/1548-8667(1994)006<0085:SANDIM>2.3.CO;2

[65] Evans JJ Klesius PH Pasnik DJ Evans JJ Klesius PH. Development of skeletal deformities in a *Streptococcus agalactiae*-challenged male Nile tilapia (*Oreochromis niloticus*) broodfish and in its offspring. Bull Eur Ass Fish Pathol. (2007) 27:151–69.

[66] Cuesta A Esteban MA. Pathologies in farmed tilapia and the use of immunostimulants and vaccines to prevent or treat diseases: An overview. In:López-Olmeda JF Sánchez-Vázquez FJ Rodrigo Fortes-Silva R, editors. Biology and Aquaculture of Tilapia. 1. First ed. Florida, US: CRC Press (2021). p. 119–36.

[67] Woo PTK Bruno DW. Diseases and Disorders of Finfish in Cage Culture. 2 ed. Wallingford, UK: Cab International (2014). 355 p.

[68] Chitmanat C Lebel P Whangchai N Promya J Lebel L. Tilapia Diseases and Management in River-Based Cage Aquaculture in Northern Thailand. Myanmar: Programme inland component – MYSAP (2016) 9–16 p.

[69] Ellis T Oidtmann B St-Hilaire S Turnbull J North B Mac- Intyre C. Fin erosion in farmed fish. In:Branson EJ, editor. Fish welfare. Oxford, UK: Blackwell Publishing Ltd (2008). p. 121–49.

[70] Ibrahim T. Diseases of Nile tilapia with special emphasis on water pollution. J Environ Sci Technol. (2019) 13:29–56. 10.3923/jest.2020.29.5634582943

[71] Nguyen VV Dong HT Senapin S Kayansamruaj P Pirarat N Rung-ruangkijkrai T. Synergistic infection of Ichthyophthirius multifiliis and Francisella noatunensis subsp orientalis in hybrid red tilapia (*Oreochromis* sp). Microb Pathog. (2020) 147:1–18. 10.1016/j.micpath.2020.104369 32634614

[72] Ali MS Griffiths D Turner WA. Practical Training Manual: Tilapia Breeding and All-Male Fry Production. Myanmar: MYSAP Inland (2020). 28 p.

[73] Mashaii N Rajabipour F. A review on the breeding of Nile tilapia, *Oreochromis niloticus* in brackish water hatchery, Iran. Int J Environ Sci Educ. (2022) 2:29–45. 10.52547/injoar.2.2.29

[74] Puttaraksar N. GIFT Technology Manual: An Aid to Tilapia Selective Breeding. Penang, Malaysia: WorldFish Center (2004). 56 p.

[75] Fessehaye Y Bovenhuis H Rezk MA Crooijmans R van Arendonk JAM Komen H. Effects of relatedness and inbreeding on reproductive success of Nile tilapia (*Oreochromis niloticus*). Aquac. (2009) 294:180–6. 10.1016/j.aquaculture.2009.06.001

[76] Fujimura K Okada N. Development of the embryo, larva and early juvenile of Nile tilapia *Oreochromis niloticus* (Pisces: Cichlidae). Developmental staging system. Dev Growth Differ. (2007) 49:301–24. 10.1111/j.1440-169X.2007.00926.x 17501907

[77] Bobe J Labbé C. Egg and sperm quality in fish. Gen Comp Endocrinol. (2010) 165:535–48. 10.1016/j.ygcen.2009.02.011 19272390

[78] Guan B Cai Y Zhou Y Zhao Z Wang Y Zhang D. Pathogen identification, risk factor and preventive measure of a columnaris disease outbreak in Tilapia (*Oreochromis niloticus*) eggs and larvae from a tilapia hatchery. Aquac. (2022) 561:738718. 10.1016/j.aquaculture.2022.738718

[79] Senapin S Dong HT Meemetta W Siriphongphaew A Charoensapsri W Santimanawong W. Hahella chejuensis is the etiological agent of a novel red egg disease in tilapia (Oreochromis spp) hatcheries in Thailand. Aquac. (2016) 454:1–7. 10.1016/j.aquaculture.2015.12.013

[80] Aich N Paul A Choudhury TG Saha H. Tilapia Lake Virus (TiLV) disease: current status of understanding. Aquac Fish. (2022) 7:7–17. 10.1016/j.aaf.2021.04.007

[81] El-Greisy ZAE-B Ahmed NAM. Effect of prolonged ammonia toxicity on fertilized eggs, hatchability and size of newly hatched larvae of Nile tilapia, *Oreochromis niloticus*. Egypt J Aquat Res. (2016) 42:215–22. 10.1016/j.ejar.2016.04.001

[82] Machimbirike VI Jansen MD Senapin S Khunrae P Rattanarojpong T Dong HT. Viral infections in tilapines: more than just tilapia lake virus. Aquac. (2019) 503:508–18. 10.1016/j.aquaculture.2019.01.03631578751

[83] Fariedadh F Widodo MS Nuswantoro S Rizal MK. Hatching of Nile tilapia (*Oreochromis niloticus*) egg in the hatching medium use salinity media and bromelain enzyme. In: IOP Conference Series: Earth and Environmental Science, Vol. 718. Surabaya (2020). 10.1088/1755-1315/718/1/012027

[84] Passos Neto OP Santos ABd Mota S. Synthetic and natural hormones impact the zootechnical and morphological characteristics of Nile tilapia (Oreochromis niloticus). Eng Sanit Ambient. (2022) 27:325–33. 10.1590/s1413-415220210098

[85] Wahbi OM Sangak Y. Enhancement of reproductive performance of nile tilapia *Oreochromis niloticus* using phytobiotic *Spirulina platensis*. J Biol Sci. (2017) 17:305–11. 10.3923/jbs.2017.305.311

[86] Fridman S Bron JE Rana KJ. Ontogenic changes in the osmoregulatory capacity of the Nile tilapia *Oreochromis niloticus* and implications for aquaculture. Aquac. (2012) 356–7:243–9. 10.1016/j.aquaculture.2012.05.010

[87] Stien LH Bracke MBM Folkedal O Nilsson J Oppedal F Torgersen T. Salmon Welfare Index Model (SWIM 10): a semantic model for overall welfare assessment of caged Atlantic salmon: Review of the selected welfare indicators and model presentation. Rev Aquac. (2013) 5:33–57. 10.1111/j.1753-5131.2012.01083.x

[88] El-Sayed A-FM Kawanna M. Effects of photoperiod on the performance of farmed Nile tilapia *Oreochromis niloticus*: I. growth, feed utilization efficiency and survival of fry and fingerlings. Aquac. (2004) 231:393–402. 10.1016/j.aquaculture.2003.11.012

[89] Pandit NP Wagle R Ranjan R. Alternative artificial incubation system for intensive fry production of Nile tilapia (*Oreochromis niloticus*). Int J Fish Aquat Stud. (2017) 5:425–9.

[90] Al Hafedh YS Siddiqui AQ Al-Saiady MY. Effects of dietary protein levels on gonad maturation, size and age at first maturity, fecundity and growth of Nile tilapia. Aquaculture International. (1999) 7:319–32. 10.1023/A:100927691136019090226

[91] Mabroke RS Tahoun AM Suloma A El-Haroun ER. Evaluation of meat and bone meal and mono-sodium phosphate as supplemental dietary phosphorus sources for broodstock Nile Tilapia (*Oreochromis niloticus*) under the conditions of hapa-in- pond system. Turk J Fish Aquat Sci. (2013) 13:02. 10.4194/1303-2712-V13_1_02

[92] Suloma A Tahoun A-A Mabroke RS. Development of brood-stock diets for nile tilapia under hapa-in-pond hatchery system; optimal dietary vitamin C level for the optimum reproductive performance and fry survival. J Aquac Res Dev. (2017) S2:010. 10.4172/2155-9546.S2-010

[93] Abdel-Tawwab M Shukry M Farrag FA El-Shafai NM Dawood MAO Abdel-Latif HMR. Dietary sodium butyrate nanoparticles enhanced growth, digestive enzyme activities, intestinal histomorphometry, and transcription of growth-related genes in Nile tilapia juveniles. Aquac. (2021) 536:736–467. 10.1016/j.aquaculture.2021.736467

[94] Ng WK Romano N A. review of the nutrition and feeding management of farmed tilapia throughout the culture cycle. Rev Aquac. (2013) 5:220–54. 10.1111/RAQ.12014

[95] Sweilum MA Abdella MM Salah El-Din SA. Effect of dietary protein-energy levels and fish initial sizes on growth rate, development and production of Nile tilapia, *Oreochromis niloticus* L. Aquac Res. (2005) 36:1414–21. 10.1111/J.1365-2109.2005.01362.X

[96] NRC. Nutrient Requirements of Fish and Shrimp. Washington, DC: National Academies Press (2011). 392 p.

[97] Gomes VDS Silva JHV Cavalcanti CR Filho JJ Almeida JLS Amâncio ALL. Avanços do uso de enzimas na nutrição de tilápias. Visão Acadêmica. (2018) 19:57380. 10.5380/acd.v19i1.57380

[98] Li C Zhao Y Wang Y Li L Yang X Chen S. Microbial community changes induced by *Pediococcus pentosaceus* improve the physicochemical properties and safety in fermented tilapia sausage. Food Res Int. (2021) 147:110476. 10.1016/j.foodres.2021.110476 34399472

[99] Kubitza F. Nutrição e alimentação dos peixes cultivados. São Paulo, Brazil: Acqua Supre (1999). 123 p.

[100] Xie S Jokumsen A. Replacement of fish meal by potato protein concentrate in diets for rainbow trout, *Oncorhynchus mykiss* (Walbaum): growth, feed utilization and body composition. Aquac Nutr. (1997) 3:65–9. 10.1046/j.1365-2095.1997.00074.x

[101] Clark AE Watanabe WO Olla BL Wicklund RI. Growth, feed conversion and protein utilization of Florida red tilapia fed isocaloric diets with different protein levels in seawater pools. Aquac. (1990) 88:75–85. 10.1016/0044-8486(90)90321-D

[102] Sanches LEF Hayashi C. Densidade de estocagem no desempenho de larvas de tilápia-do-Nilo (Oreochromis niloticus L), durante a reversão sexual. Acta Sci. (1999) 21:619–25. 10.4025/actascianimsci.v21i0.4299

[103] Bombardelli RA Hayashi C Natali MRM Sanches EA Piana PA. Níveis de energia digestível sobre os desempenhos reprodutivo e zootécnico e a deposição de lipídios nos hepatócitos de machos de tilápia-do-nilo. Rev Bras Zootec. (2010) 39:941–9. 10.1590/S1516-35982010000500001

[104] Chowdhury DK. Optimal Feeding Rate for Nile Tilapia (Oreochromis niloticus) (PhD thesis). Norwegian: Norwegian University of Life Sciences (2011).

[105] Bhujel RC Little DC Hossain A. Reproductive performance and the growth of pre-stunted and normal Nile tilapia (*Oreochromis niloticus*) broodfish at varying feeding rates. Aquac. (2007) 273:71–9. 10.1016/j.aquaculture.2007.09.022

[106] Venturini FP Vargas Baldi SC Parisi G Costa TD Rucinque DS Pires Melo M. Effects of different stunning methods on blood markers and enzymatic activity of stress responses of tilapia (*Oreochromis niloticus*). Ital J Anim Sci. (2018) 17:1094–8. 10.1080/1828051X.2018.1426396

[107] Santiago CB. Nutrition and feeds of Nile tilapia broodstock and fry. In:Fortes RD Darvin LC de Guzman DL, editors. Fish and Crustacean Feeds and Nutrition?: Proceedings of the Seminar-Workshop On Fish And Crustacean Feeds and Nutrition Held on 25-26 February 1985 at UPV, Iloilo City. Laguna: Philippine Council for Aquatic and Marine Research and Development (1989). p. 40–9.

[108] Malik Daudpota A Abbas G Bux Kalhoro I Sajjad Shah SA Kalhoro H Hafeez-ur-Rehman M. Effect of feeding frequency on growth performance, feed utilization and body composition of juvenile Nile Tilapia, Oreochromis niloticus (L) reared in low salinity water. Pakistan J Zool. (2016) 48:171–7.

[109] Ferdous Z Nahar N Hossen MS Sumi KR Ali MM. Performance of different feeding frequency on growth indices and survival of monosex tilapia, *Oreochromis niloticus* (Teleostei: Cichlidae) fry. Int J Fish Aquat Stud. (2014) 1:80–3.

[110] Garcia JA Villarroel M. Effect of feed type and feeding frequency on macrophage functions in tilapia (Oreochromis niloticus L.) *Fish Shellfish Immunol*. (2009) 27:325–9. 10.1016/J.FSI.2009.05.018 19501652

[111] Riche M Haley D Oetker M Garbrecht S Garling D. Effect of feeding frequency on gastric evacuation and the return of appetite in tilapia Oreochromis niloticus (L.) *Aquac*. (2004) 234:657–73. 10.1016/j.aquaculture.2003.12.012

[112] Morgan IJ Metcalfe NB. Deferred costs of compensatory growth after autumnal food shortage in juvenile salmon. Proc R Soc B: Biol Sci. (2001) 268:295–301. 10.1098/rspb.2000.1365 11217901 PMC1088606

[113] Jobling M Johansen S Foshaug H Burkow I Jørgensen E. Lipid dynamics in anadromous Arctic charr, Salvelinus alpinus (L): seasonal variations in lipid storage depots and lipid class composition. Fish Physiol Biochem. (1998) 18:225–40. 10.1023/A:1007747201521

[114] Lovell T. Nutrition and Feeding of Fish. Alabama, US: Springer New York, NY (1989). 262 p.

[115] Tacon AGJ Metian M. Feed Matters: Satisfying the Feed Demand of Aquaculture. Rev Fish Sci Aquac. (2015) 23:1–10. 10.1080/23308249.2014.987209

[116] Borges AM. Criação de tilápias. Sette NMdC, editor. Brasília, DF: Emater (2009). 44 p.

[117] Marking LL Meyer FP. Are better anesthetics needed in fisheries? Fisheries. (1985) 10:2–5. 10.1577/1548-8446(1985)010<0002:abanif>2.0.co;235460418

[118] Ross LG Ross B. Anaesthetic and Sedative Techniques for. 3 ed. Oxford, UK: Blackwell (2008). 240 p.

[119] Noble C Gismervik K Iversen MH Kolarevic J Nilsson J Stien LH. Welfare Indicators for Farmed Atlantic Salmon: Tools for Assessing Fish Welfare. Norway: Nofima (2018). 352 p.

[120] Davis MW. Fish stress and mortality can be predicted using reflex impairment. Fish Fish. (2010) 11:1–11. 10.1111/j.1467-2979.2009.00331.x

[121] Robb D Kestin SC. Methods used to kill fish: field observations and literature reviewed. Anim Welf. (2002) 11:269–82. 10.1017/S096272860002485424716788

[122] Müller-Graf C Berthe F Grudnik T Peeler E Afonso A. Risk assessment in fish welfare, applications and limitations. Fish Physiol Biochem. (2012) 38:231–41. 10.1007/s10695-011-9520-1 21671027

[123] IGN. Fish Welfare in Aquaculture–Problems and Approaches. Munich, Germany: International Society of Livestock Husbandry (2020). 96 p.

[124] Foster C. What a fish knows: the inner lives of our underwater cousins by Jonathan Balcombe. Common Knowle. (2018) 24:315–6.

[125] Saraiva JL Arechavala-Lopez P Castanheira MF Volstorf J Heinzpeter Studer B. A global assessment of welfare in farmed fishes: the FishEthoBase. Fishes. (2019) 4:30. 10.3390/fishes4020030

[126] Flores-García L Camargo-Castellanos JC Pascual-Jímenez C Almazán-Rueda P Monroy-López JF Albertos-Alpuche PJ. Welfare indicators in tilapia: an epidemiological approach. Front Vet Sci. (2022) 9:882567. 10.3389/fvets.2022.882567 35832331 PMC9271997

[127] Norris IM. Identification of Potential Welfare Indicators for Commercially Farmed King Salmon (Hāmana, Oncorhynchus tshawytscha): a Scoping Review to Inform the Development of a National Code of Welfare (dissertation/master's thesis). Manawatu, New Zealand: Massey University (2022)..

[128] Conte FS. Stress and the welfare of cultured fish. Appl Anim Behav Sci. (2004) 86:205–23. 10.1016/j.applanim.2004.02.003

[129] Broom DM. Animal welfare: concepts and measurement. J Anim Sci. (1991) 69:4167–75. 10.2527/1991.69104167x 1778832

[130] Whittaker AL Golder-Dewar B Triggs JL Sherwen SL McLelland DJ. Identification of animal-based welfare indicators in captive reptiles: a delphi consultation survey. Animals. (2021) 11:2010. 10.3390/ani11072010 34359138 PMC8300299

[131] Raposo d Magalhães C Schrama D Farinha AP Revets D Kuehn A Planchon S. Protein changes as robust signatures of fish chronic stress: a proteomics approach to fish welfare research. BMC genom. (2020) 21:1–16. 10.1186/s12864-020-6728-4 32306896 PMC7168993

[132] Afonso LO. Identifying and managing maladaptive physiological responses to aquaculture stressors. In:Tillmann J. Benfey APF Colin J. Brauner, editor. Fish Physiology. Amsterdam: Elsevier (2020). p. 163–91.

[133] Gesto M. Characterization of the neuroendocrine stress status as part of the multiparametric assessment of welfare in fish. In:Monzón IF Fernandes JMO, editors. Cellular and Molecular Approaches in Fish Biology. Amsterdam: Elsevier (2022). p. 285–308.

[134] Dara M. Fish Welfare in Aquaculture: From Physiology to Molecular Activities and New Tools For Study Innovative Diets, Social and Spatial Stress (PhD thesis). Palermo, Italy: Università degli Studi di Palermo (2022).

[135] Swirplies F Wuertz S Baßmann B Orban A Schäfer N Brunner RM. Identification of molecular stress indicators in pikeperch Sander lucioperca correlating with rising water temperatures. Aquac. (2019) 501:260–71. 10.1016/j.aquaculture.2018.11.043

[136] Sejian V Lakritz J Ezeji T Lal R. Assessment methods and indicators of animal welfare. Asian J Anim Vet Adv. (2011) 6:301–15. 10.3923/ajava.2011.301.315

[137] Wemelsfelder F Mullan S. Applying ethological and health indicators to practical animal welfare assessment. Rev Sci Tech. (2014) 33:111–20. 10.20506/rst.33.1.2259 25000783

[138] Rousing T Bonde M Sørensen JT. Aggregating welfare indicators into an operational welfare assessment system: a bottom-up approach. Acta Agric Scand A Anim Sci. (2001) 51:53–7. 10.1080/090647001300004790

[139] Saberioon M Gholizadeh A Cisar P Pautsina A Urban J. Application of machine vision systems in aquaculture with emphasis on fish: state-of-the-art and key issues. Rev Aquac. (2017) 9:369–87. 10.1111/raq.12143

[140] Pettersen JM Bracke MBM Midtlyng PJ Folkedal O Stien LH Steffenak H. Salmon welfare index model 20: An extended model for overall welfare assessment of caged Atlantic salmon, based on a review of selected welfare indicators and intended for fish health professionals. Rev Aquac. (2014) 6:162–79. 10.1111/raq.12039

[141] Arechavala-Lopez P Cabrera-Álvarez MJ Maia CM Saraiva JL. Environmental enrichment in fish aquaculture: a review of fundamental and practical aspects. Rev Aquac. (2022) 14:704–28. 10.1111/raq.12620

[142] Bui S Oppedal F Sievers M Dempster T. Behaviour in the toolbox to outsmart parasites and improve fish welfare in aquaculture. Rev Aquac. (2019) 11:168–86. 10.1111/raq.12232

[143] Carbonara P Alfonso S Gai F Gasco L Palmegiano G Spedicato MT. Moderate stocking density does not influence the behavioural and physiological responses of rainbow trout (*Oncorhynchus mykiss*) in organic aquaculture. Aquac Res. (2020) 51:3007–16. 10.1111/are.14640

[144] Martos-Sitcha JA Prunet P Mancera JM Magnoni LJ. Welfare and stressors in fish: challenges facing aquaculture. Front Physiol. (2020) 11:199. 10.3389/fphys.2020.00162 32174844 PMC7055460

[145] Saraiva JL Rachinas-Lopes P Arechavala-Lopez P. Finding the “golden stocking density”: a balance between fish welfare and farmers' perspectives. Front Vet Sci. (2022) 9:930221. 10.3389/fvets.2022.930221

[146] MacKinnon B Debnath PP Bondad-Reantaso MG Fridman S Bin H Nekouei O. Improving tilapia biosecurity through a value chain approach. Rev Aquac. (2023) 15:57–91. 10.1111/raq.12776

[147] Shlapobersky M Sinyakov MS Katzenellenbogen M Sarid R Don J Avtalion RR. Viral encephalitis of tilapia larvae: primary characterization of a novel herpes-like virus. Virology. (2010) 399:239–47. 10.1016/j.virol.2010.01.001 20117816

[148] Bhujel R Perera A. Shading of breeding hapas enhances reproductive performance of nile tilapia (*Oreochromis niloticus*) and seed output. J Aqua Trop. (2018) 32:187–97.

[149] Dayrit GB Vera Cruz EM Rodkhum C Mabrok M Ponza P Santos MD. Potential influence of shading in freshwater ponds on the water quality parameters and the hematological and biochemical profiles of Nile tilapia (*Oreochromis niloticus* Linnaeus, 1758). Fishes. (2023) 8:322. 10.3390/fishes8060322

[150] Saraiva JL Nogueirinha M Teodósio R Aragão C Engrola S Arechavala-Lopez P. The effect of tank cover on welfare of farmed Nile tilapia. Appl Anim Behav Sci. (2021) 241:105396. 10.1016/j.applanim.2021.105396

[151] Napoli MAS. (2015) *Consumo de água na industrialização da Tilápia: estudo de caso do oeste do Paraná* (dissertation/master's thesis). Paraná, Brazil: Universidade Estadual do Oeste do Paraná.

[152] Coelho M Pedrazzani A Quintiliano M Bolfe F Molento C. Fish slaughter practices in Brazilian aquaculture and their consequences for animal welfare. Anim Welf. (2022) 31:187–92. 10.7120/09627286.31.2.003

[153] Hossain MD Jónsson Á. (2013) *Effect of Combined Blast and Contact (cbc) Cooling and Gutting on the Quality of Tilapia (Oreochromis niloticus) during Chilled Storage* (dissertation/master's thesis). Reykjavík, Iceland: United Nations University Fisheries Training Programme.

[154] Eltholth M Fornace K Grace D Rushton J Häsler B. Characterisation of production, marketing and consumption patterns of farmed tilapia in the Nile Delta of Egypt. Food Policy. (2015) 51:131–43. 10.1016/j.foodpol.2015.01.002

[155] Lines JA Spence J. Humane harvesting and slaughter of farmed fish. Rev Sci Tech. (2014) 33:255–64. 10.20506/rst.33.1.2284 25000798

[156] NFDB. Guidelines for responsible farming of tilapia in India. In: Department of Animal Husbandry DF, Ministry of Agricultura and Farmers Welfare, Govt. of lndia. New Delhi, India: National Fisheries Development Board (2015). p. 16.

[157] Nasr-Allah A Dickson M Al-Kenawy D Ahmed Ibrahim N Ali S Karisa H. Better Management Practices for Tilapia Culture in Egypt. Penang, Malaysia: CGIAR Research Program on Fish Agri-Food Systems (2021). 30 p.

[158] Gonçalves-De-Freitas E Bolognesi MC Gauy ACDS Brandão ML Giaquinto PC Fernandes-Castilho M. Social behavior and welfare in Nile Tilapia. Fishes. (2019) 4:1–14. 10.3390/fishes4020023

[159] Toguyeni A Fauconneau B Boujard T Fostier A Kuhn ER Mol KA. Feeding behaviour and food utilisation in tilapia, *Oreochromis niloticus*: effect of sex ratio and relationship with the endocrine status. Physiol Behav. (1997) 62:273–9. 10.1016/S0031-9384(97)00114-5 9251968

[160] El-Sayed A-FM Kawanna M. Effects of photoperiod on growth and spawning efficiency of Nile tilapia (Oreochromis niloticus L) broodstock in a recycling system. Aquac Res. (2007) 38:1242–7. 10.1111/j.1365-2109.2007.01690.x

[161] Campos-Mendoza A McAndrew BJ Coward K Bromage N. Reproductive response of Nile tilapia (*Oreochromis niloticus*) to photoperiodic manipulation; effects on spawning periodicity, fecundity and egg size. Aquac. (2004) 231:299–314. 10.1016/j.aquaculture.2003.10.023

[162] Ramos J Balasch JC Tort L. About welfare and stress in the early stages of fish. Front Vet Sci. (2021) 8:634434. 10.3389/fvets.2021.634434 33693043 PMC7937697

[163] Labbé C Robles V Herraez MP. Epigenetics in fish gametes and early embryo. Aquac. (2017) 472:93–106. 10.1016/j.aquaculture.2016.07.026

[164] Lopez-Luna J Al-Jubouri Q Al-Nuaimy W Sneddon LU. Reduction in activity by noxious chemical stimulation is ameliorated by immersion in analgesic drugs in zebrafish. J Exp Biol. (2017) 220:1451–8. 10.1242/jeb.146969 28424313

[165] Wilson C. Aspects of larval rearing. ILAR Journal. (2012) 53:169–78. 10.1093/ilar.53.2.169 23382348

[166] Bordeleau X Pardo S Chaput G April J Dempson B Robertson M. Spatio-temporal trends in the importance of iteroparity across Atlantic salmon populations of the northwest Atlantic. ICES J Mar Sci. (2020) 77:326–44. 10.1093/icesjms/fsz188

[167] Cuevas-Rodríguez BL García-Ulloa M Hernández-Llamas A Racotta I Valdez-González FJ Polanco-Torres A. Evaluating quality of Nile tilapia (*Oreochromis niloticus*) eggs and juveniles from different commercial hatcheries. Lat Am J Aquat Res. (2017) 45:213–7. 10.3856/vol45-issue1-fulltext-23

[168] Birch J. Animal sentience and the precautionary principle. Anim Sentience. (2017) 16:1200. 10.51291/2377-7478.1200

[169] Engle CR van Senten J Clark C Boldt N. Has the regulatory compliance burden reduced competitiveness of the US tilapia industry? Fishes. (2023) 8:151. 10.3390/fishes8030151

[170] Ellis T Berrill I Lines J Turnbull JF Knowles TG. Mortality and fish welfare. Fish Physiol Biochem. (2012) 38:189–99. 10.1007/s10695-011-9547-3 21922247

[171] Tallentire CW Edwards SA Van Limbergen T Kyriazakis I. The challenge of incorporating animal welfare in a social life cycle assessment model of European chicken production. Int J Life Cycle Assess. (2019) 24:1093–104. 10.1007/s11367-018-1565-2

[172] Folkedal O Pettersen J Bracke M Stien L Nilsson J Martins C. On-farm evaluation of the Salmon Welfare Index Model (SWIM 10): theoretical and practical considerations. Anim Welf. (2016) 25:135–49. 10.7120/09627286.25.1.135

[173] Bartlett H Balmford A Holmes MA Wood JL. Advancing the quantitative characterization of farm animal welfare. Proc R Soc B: Biol Sci. (2023) 290:20230120. 10.1098/rspb.2023.0120 36946112 PMC10031399

[174] Clark B Stewart GB Panzone LA Kyriazakis I Frewer LJ. A systematic review of public attitudes, perceptions and behaviours towards production diseases associated with farm animal welfare. J Agric Environ Ethics. (2016) 29:455–78. 10.1007/s10806-016-9615-x

[175] Peng W Robinson BE Zheng H Li C Wang F Li R. The limits of livelihood diversification and sustainable household well-being, evidence from China. Environ Dev. (2022) 43:100736. 10.1016/j.envdev.2022.100736

