# Supplementary figures and images for: From egg to slaughter: monitoring the welfare of Nile tilapia, Oreochromis niloticus, throughout their entire life cycle in aquaculture
Source: Front Vet Sci. 2023 Sep 21;10:1268396. doi: 10.3389/fvets.2023.1268396 (PMC10551173; doi:10.3389/fvets.2023.1268396)

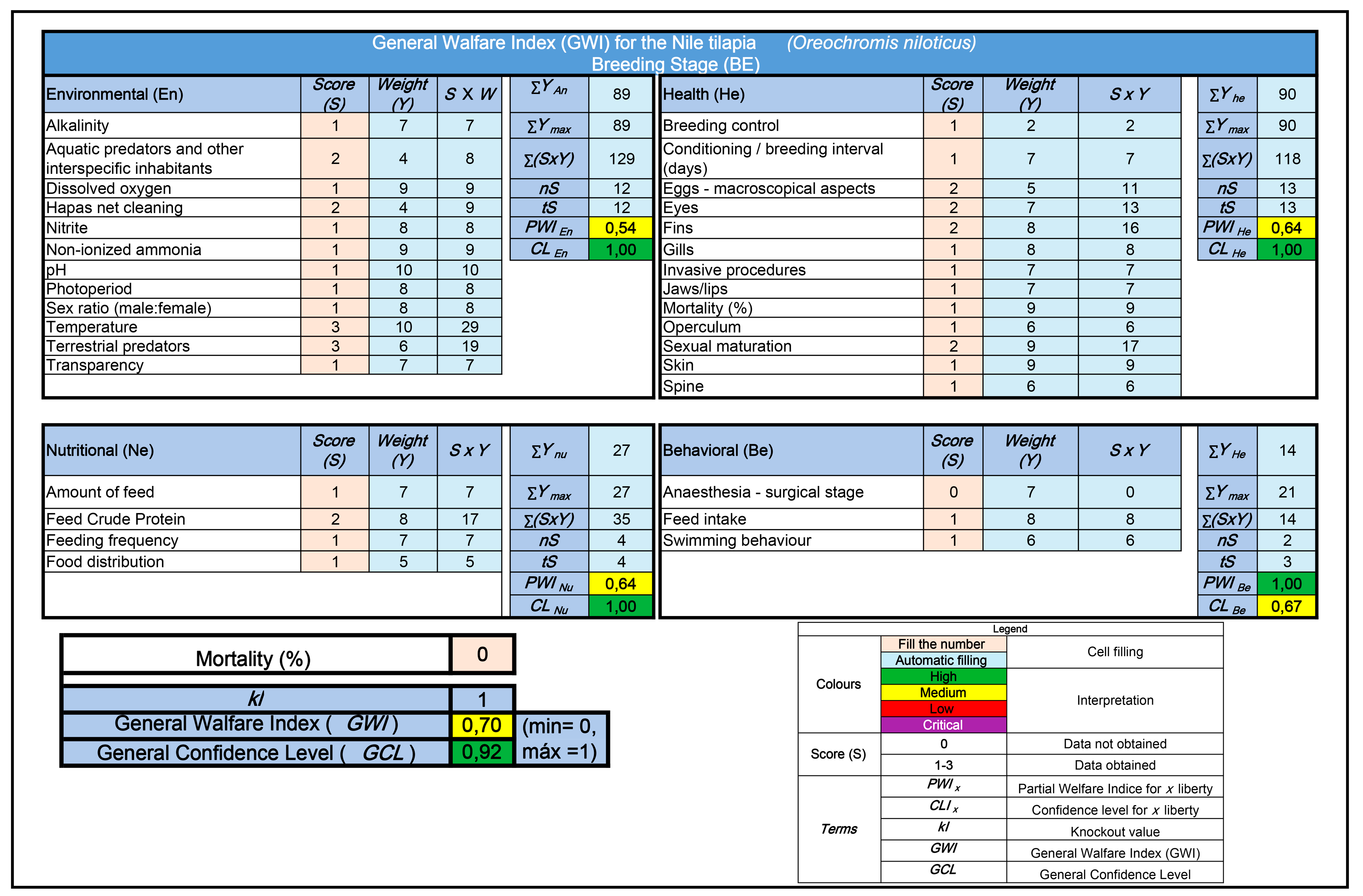

Supplement: Supplementary file 1 [file Image_1.PNG]

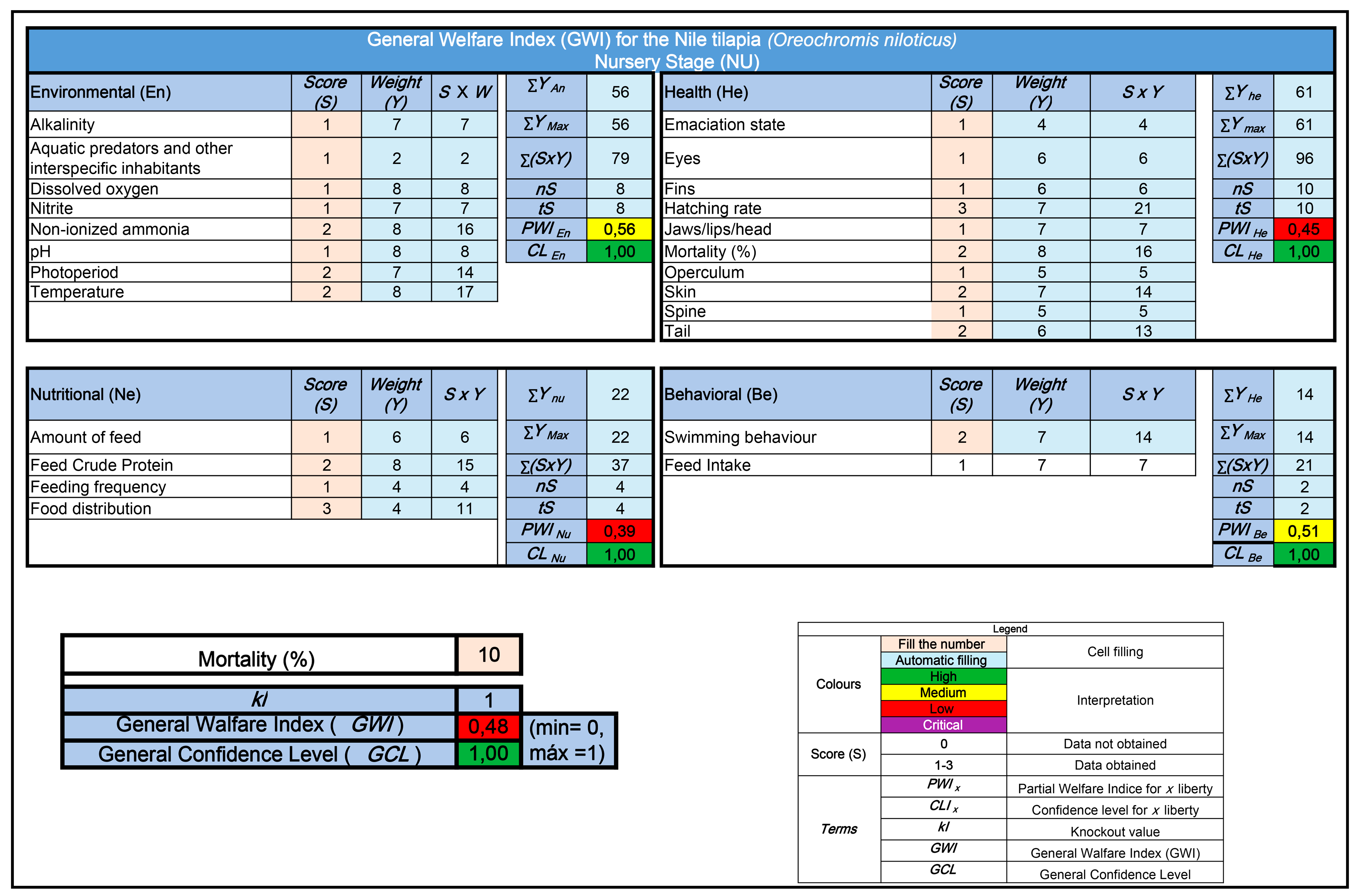

Supplement: Supplementary file 2 [file Image_2.PNG]
